# An intelligent hybrid approach combining fuzzy C-means and the sperm whale algorithm for cyber attack detection in IoT networks

**DOI:** 10.1038/s41598-024-79230-4

**Published:** 2025-01-06

**Authors:** E. I. Elsedimy, Sara M. M. AboHashish

**Affiliations:** Department of Information Technology Management, Faculty of Management Technology and Information System, Port Said University, Port Said, 42526 Egypt

**Keywords:** Internet of things, Smart cities, Fuzzy C-means, Sperm whales algorithm, Engineering, Mathematics and computing

## Abstract

The Internet of Things (IoTs) has revolutionized cities, enabling them to become smarter. IoTs play an important role in monitoring the traffic cameras, roads, smart farming, connected vehicles, air quality, water level, humidity, and carbon dioxide pollution levels in city buildings. One of the major challenges of smart cities is the cyber threat to sensitive data. This paper presents an intelligent approach for detecting cyberattacks and mitigating malicious events in IoT-based smart systems. The proposed approach, known as FCM-SWA, hybridizes a fuzzy C-mean (FCM) with a sperm whale algorithm (SWA). In the first step, we use a novel SWA optimization algorithm to enhance the FCM performance and provide effective defenses against various types of smart city attacks. Next, we propose an adaptive threshold strategy to enhance the global search capability of SWA and prevent the algorithm from settling into local optima. Lastly, we present an efficient scaling approach that solves the clustering problem and finds the optimal cluster center, striking a balance between exploration and exploration in the search space. The proposed FCM-SWA model does better than related and state-of-the-art methods in terms of accuracy, detection rate, precision rate, and F1-scores, as shown by experiments on the NSL-KDD, AWID, and BoT-IoT datasets.

## Introduction

**Fig. 1:**
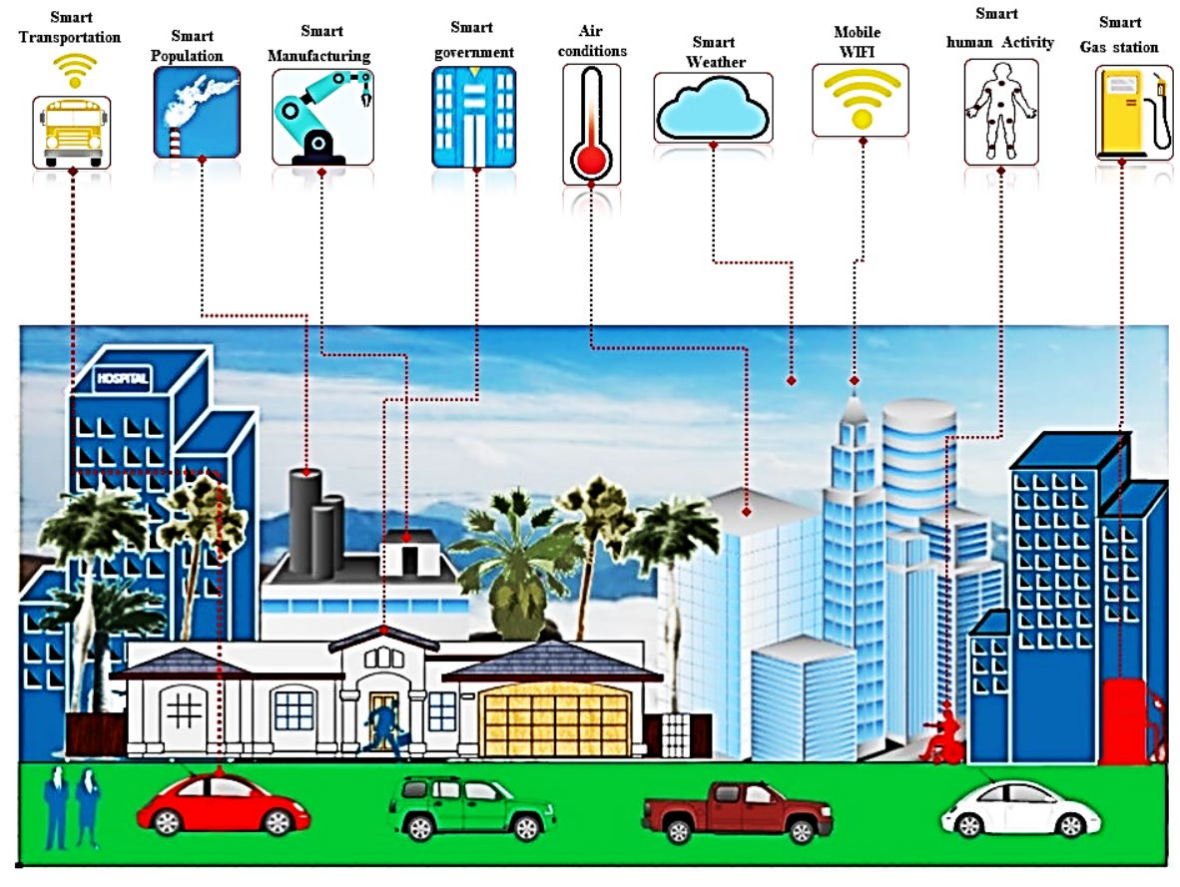
IoT systems in smart cities.

The Internet of Things (IoT) has significantly expanded its applications in business, home automation, healthcare, agriculture, and other global services^1^. Any aspect of human life can benefit from the application of IoT. One can interconnect IOT devices to control and monitor environmental conditions in a weather station^2^, smart cities^3^, banking systems^4^, agro-industry and environment^5^, human activity recognition^6^, and others^7,8^. A new report from Allied Market Research predicts that the market for IoT devices, which earned $77.8 billion in 2021, will grow at a CAGR of 18.6% from 2022 to 2031, reaching $413.7 billion. Furthermore, Allied Market Research predicts that the number of connected IoT devices will increase from 27 billion in 2017 to over 125 billion by 2030^9^. Figure 1 illustrates a variety of smart city services, including climate-smart agriculture systems that address food security and combat climate change, monitoring systems that monitor temperature, humidity, and carbon dioxide pollution levels in city structures, smart health settings in hospitals that monitor patients for therapeutic purposes, smart parking services that use road sensors and sophisticated displays to guide cars where to park in the city, and smart lighting that adjusts street lamp intensity based on the time of day, the weather, and the presence of people^10^. Many other fields, particularly smart cities, use IOT, making the data gathered from these systems a significant source of big data. It is evident that smart cities, due to their large number of sensors, are particularly costly in terms of data, with one of the primary objectives being to filter and control large sensing data streams to prevent attacks. Besides, IoT systems in smart cities use unprotected default keys, leaving them vulnerable to hacking and exploitation. In addition, the resource limitations of IoT systems, specifically their battery power, limit the implementation of security solutions on the actual devices. Therefore, it is essential to provide smart cities with effective defenses against a variety of attack types. To that end, one of the most important and difficult issues in the world is automated attack prediction.

Attackers use a variety of hacking techniques to compromise unprotected, unpatched, or unencrypted IoT devices to achieve their objectives, such as stealing sensitive data and polluting IoT resources. This poses a significant threat to the sustainability of smart cities. Attacks on IoT devices not only prevent access to these services but also put the field of green IoT cities, which enables energy-efficient services in the larger IoT ecosystem, at risk. In order to get access to IoT systems and associated networks, attackers used a number of botnet techniques, including reconnaissance, malicious control, spying, and denial of service (DoS) attacks on IoT devices with insufficient protection. Kareem, Mostafa, Hashim, and El-Bakry^11^ presented a novel IoT IDS technique to overcome this. With their proposed technique, the authors were able to identify malicious actions like port scanning and brute-force attacks. Qureshi, Larijani, Ahmad, and Mtetwa^12^ have proposed anomaly-based IDS, a model similar to this one. Their technology accurately found DoS attacks and SQL injection vulnerabilities in IoT applications. The authors tested their proposed method on the NSL-KDD dataset and found that their model had a 95.25% success rate in identifying attacks. Besides, Ali, Al Mohammed, Ismail, and Zolkipli^13^ presented a rapid learning network based on particle swarm optimization (PSO) and other techniques. The authors tested their proposed technique using the KDD-99 dataset. The accuracy of the proposed model’s attack prediction was 98.92%. Even though their model performed well, its complexity makes it unsuitable for devices with limited resources. Consequently, it is extremely difficult to detect malicious behavior without first extracting relevant and usable data elements. One solution for these smart cities is to use modern machine learning models, which will instantly combine the collected data from the IoT network and evaluate the extracted data to identify the hidden malicious software^14^.

The application of machine learning models for cyberattack detection in smart cities has grown rapidly during the past few years. They play a vital role in identifying attacks in the IoT ecosystem^15^. For example, Hasan et al.^16^ presented a comprehensive data analysis technique for anomaly detection at IoT sites. The proposed method evaluated various machine learning approaches against the DS2OS dataset. Although these approaches are equally accurate, random forest (RF) performs much better based on other metrics. In the same context, Liu et al. developed a joint trust light probe-based protection (TLPD) method for detecting on-and-off attacks in an IoT network^17^. However, it cannot detect any new malicious behavior on the IoT network. In addition, Pajouh et al. employed Nave Bayes (NB) and K-Nearest Neighbor (KNN) techniques to detect suspicious activity at IoT sites^18^. They used principle component analysis (PCA) and linear discriminate analysis (LDA) to reduce the number of features in the NSL-KDD dataset, improving accuracy to 84.82%. Latah and Toker^19^ also proposed an anomaly-based intrusion detection model for a centralized network architecture. This framework evaluated numerous machine learning strategies. In their results, the decision tree achieved the greatest rate of accuracy (88.74%). Furthermore, Pahl and Aubet^20^ validated their use of K-means and BIRCH-based clustering for IDS on IoT locations using DS2OS data. The overall accuracy of the proposed method is about 96.3%. However, a local minimum traps the classic K-means approach. In general, these state-of-the-art models are computationally costly and tend to converge on local minima. Therefore, an improved machine learning model based on a global search algorithm is required to address these challenges.

The detection of cyberattack events within Industrial Internet Control Systems (IICS) is becoming more challenging for intrusion detection systems (IDS) due to the ever-increasing number of devices in the Industrial Internet of Things (IIoT)^21^. Ahmed et al.^21^ used the UNSW-NB15 and gas pipeline datasets to train an LSTM-based deep autoencoder. To successfully identify intrusive actions in IICS networks, the authors employed the LSTM architecture inside the autoencoder. As a result, the gas pipeline achieved a 97.95% accuracy rate and UNSW-NB15 a 97.62% accuracy rate. In the same context, Ahmed et al.^22^ proposed a novel deep learning approach in the same area. To improve the data feature learning, they use a two-stage sliding window (SW) method. The first thing that the SW does is convert the raw time series into fixed-length sequences by removing any harmful points. Then, to help the model better capture hidden representations of harmful actions, they use a smaller SW to split each sequence into continuous pieces. Once the traits have been recovered, the fully connected networks can use them to categorize and explain attack occurrences. In order to enhance security in IoTs settings with constrained resources and gas pipeline datasets, Ahmed et al.^23^ has developed a novel class of intrusion detection systems known as federated-simple recurrent units (SRUs). The suggested model addressed the gradient vanishing problem in ICS networks and reduced computing overhead by utilizing SRU architecture. With federated learning, all ICS devices can process data locally without transmitting private information to a server. Besides, Elsedimy et al.^24^ secured the IoT network by combining the algorithms of Quantum Support Vector Machine (QSVM) and Improved Grey Wolf Optimizer (IGWO). In order to improve classification accuracy and decrease computation time, QSVM employs quantum kernels to transform data into a quantum feature. The IGWO algorithm improves the search procedure and allows for more efficient exploration and exploitation in the optimization process by utilizing the concept of social hierarchy in wolf packs. The results also show that the suggested model did very well on the Bot-IoT dataset, with an accuracy of 96.09 and an F1-score of 96.78. Khan et al.^25^ used a variety of machine learning models to identify assaults in healthcare IoT, such as boosting, adaptive boosting, perceptron, and RF. For binary and multiclass classification, they employed RF. Its 99.55% accuracy was obtained by addressing seven primary types of Internet of Things attacks.

 Among machine learning models, fuzzy C-means (FCM)^26^ has demonstrated successful performance in a variety of clustering and prediction problems across a diversity of areas of study. FCM is the most popular technique for unsupervised machine learning, in which unlabeled dataset elements are clustered based on their degree of similarity. Unsupervised learning, on the other hand, has worse classification accuracy because humans must manually give cluster numbers. To address this issue, a semi-supervised machine learning technique that combines supervised and unsupervised machine learning approaches has been proposed. Some of the current real-life applications of the FCM technique include image segmentation, manufacturing processes, automated speech, financial time-series forecasting, and cybercrime detection. The performance and behavior of FCM are strongly dependent on their hyperparameter values^27^. Hence, selecting the proper FCM hyper-parameter values for a particular dataset is challenging. In recent years, several studies have formulated the problem of selecting FCM hyperparameter values as an optimization problem, which gives superior outcomes compared to conventional models. Therefore, a global search-based metaheuristic intelligence method is required to improve the performance of FCM.

Several optimization techniques have been proposed to address difficulties in wireless sensor networks, including a novel meta-heuristic optimization method termed “Sperm Swarm Optimization (SSO),” which is inspired by the mobility of sperm during fertilization^28^. SSO transports sperm from the cervix to the fallopian tubes, facilitating optimum fertilization. It enhances the quality of wireless sensor networks to reduce delay, latency, packet throughput, and energy consumption. Additionally, a new meta-heuristic optimization technique, inspired by the Chernobyl nuclear reactor catastrophe, referred to as the Chernobyl Disaster Optimizer (CDO), is introduced in^29^. CDO emulates the process of nuclear radiation affecting individuals’ post-explosion, involving gamma, beta, and alpha particles. The authors assess the CDO utilizing the CEC 2017 test bed suites and juxtapose it with established optimization techniques such as Particle Swarm Optimization and Gravitational Search Algorithm, illustrating its efficacy as a plausible alternative.

The Sperm Whale Algorithm (SWA) is a new population-based meta-heuristic optimization technique that can be used to detect cyberattacks in IoT networks^30^. The SWA algorithm’s search mimics the hunting habits of sperm whales. The algorithm divides the population into smaller groups, ensuring a uniform distribution of superior and inferior solutions. We utilize the positions of the leader and the worst member of each group to calculate an intermediate distance. The goal of SWA is to find the best possible solution to the optimization problem in a fixed number of iterations. SWA is less computationally expensive and can provide a user-friendly optimization model for a variety of applications compared to many other meta-heuristic methods that need extensive parameter tuning^31^. SWA has the ability to balance exploration and exploitation effectively, as well as its simplicity in implementation. On the other hand, it has performance limitations in highly complex search spaces and the need for further improvements in specific applications. Thus, this research uses SWA to identify the optimal parameters of fuzzy C-means in order to accurately predict cyber-attacks in smart cities.

The motivations and contributions of this study stem from the growing susceptibility of Internet of Things (IoT) environments to cyberattacks, which pose significant security threats due to the interconnected nature of IoT devices. The study specifically designs the novel hybrid model FCM-SWA for effective cyber-attack detection in IoT systems, aiming to address these challenges. The FCM-SWA model combines fuzzy C-means (FCM) clustering with the Social Welfare Algorithm (SWA). The best parts of both methods are used to improve threat detection accuracy and speed. By integrating these methods, the proposed model aims to improve detection rates, reduce false positives, and provide a robust framework for securing IoT infrastructures. This contribution is crucial for advancing IoT security and ensuring the safe and reliable operation of smart devices in various applications. We evaluated the proposed model’s performance using three datasets: NSL-KDD^32^, Aegean wifi intrusion dataset (AWID)^33^, and BoT-IoT^34^. The FCM-SWA model performed better than other models due to the balance between exploration and exploitation in the solution search space. The process starts with data preparation, which involves cleaning all training datasets to extract features that are relevant to decision-making. Following this, the SWA initiates an initialization phase that generates random hyper-parameters for the FCM-SWA and calculates their training accuracy. Finally, FCM-SWA uses the SWA algorithm for tuning the FCM-SWA parameters and avoids dropping into local minima during the running stage. The procedure persists until the termination condition is satisfied.

The key contributions of this paper can be summarized as follows:

- We proposed a novel cyber-attack detection model that integrates a semi-supervised fuzzy C-means with the SWA algorithm to support efficient attack detection in IoT networks in smart cities.
- We develop an adaptive threshold strategy to enhance the global search ability of SWA and prevent the algorithm to stuck in local minima.
- We measure the F1-Score, accuracy, detection rate, and precision rate of the proposed FCM-SWA model using three benchmark IoT datasets generated by IoT devices.

This paper organizes the remaining sections as follows: Sect. 2 describes the maximum correntropy criterion method and the Sperm Whale algorithm. Section 3 outlines the proposed FCM-SWA approach for detecting cyberattacks in IoT networks. Section 4 discusses the experimental results. Section 5 concludes the paper with a summary of future research directions.

## Related work

Intrusion Detection Systems (IDS) are classified into host-based IDS and network-based IDS. Several research articles use IDSs to detect botnet attacks in smart cities^35^. However, these studies often overlook the need for IoT device resources to train their models. The high cost of such models prevents their training on a low-cost gateway device. Additionally, the offline training phase renders the model unreliable. Furthermore, a number of factors contribute to the ineffectiveness of host-based IDS in identifying hacked IoT devices^36,37^. The first factor to consider is the time and power consumption of the smart IoT device’s detecting algorithms. Secondly, certain IoT devices have restrictions on the types of software they can load. Finally, smart cities have a lot of different kinds of IoT devices, making it difficult to install sophisticated IDS on all of them. Therefore, researchers have conducted numerous studies to address the performance limitations of IDS. Recently, Vinayakumar et al. have published a two-tiered deep learning approach for identifying botnets^38^. The researchers were able to differentiate between attacks and regular traffic. Their results showed that their proposed system was more efficient in terms of accuracy, F1 score, and detection speed. On the other hand, Tuan et al. used different machine learning techniques, such as support vector machines (SVM), decision trees (DT), neural-based (NB), artificial neural networks (ANN), and K-means, to find distributed denial of service (DDoS) attacks. All of the proposed models were able to accurately distinguish between malicious botnet traffic and benign network traffic. However, the ANN model was unable to correctly recognize DoS attacks due to its total flatness. Table 1 presents a comprehensive overview of related works on cyber-attack detection strategies in IoT networks, showcasing various approaches and methodologies proposed by different researchers. The table shows the variety of methods that were used, from those based on machine learning to those based on heuristics and rules. Each of these methods dealt with a different problem that came up because IoT environments are so complex and different. Additionally, it outlines the strengths and weaknesses of each method, offering valuable insights into their detection accuracy, computational efficiency, and adaptability to evolving attack vectors. This summary serves as a foundation for understanding the current landscape of cybersecurity in IoT networks and identifying potential avenues for future research.

Zhou and Cheng used a combination of the Bat algorithm and correlation-based feature selection (CFS) to find the best features for their proposed IDS model^39^. The accuracy of the experiments on the NSL-KDD, AWID, and CIC-IDS2017 datasets was 99.8%, 99.5%, and 99.8%, respectively. In addition, Diro and Chilamkurti created a deep learning model for detecting DDoS in IoT networks. The suggested model achieves superior results in comparison to a four-class shallow neural network (normal, DoS, Probe, R2L, and U2R). It obtains 99.2% for binary-class identification and 98.27% for multi-class identification. However, their research tested the suggested model using just the NSL-KDD dataset, ignoring other well-known IoT cyber-attack datasets such as the Aegean WiFi intrusion dataset (AWID) and BoT-IoT. Recently, Wang et al.^40^ developed a novel feature-selection-based intrusion detection system (IDS) that uses a multilayer perception (MLP) classifier to find DDoS attacks. The sequential feature selection technique produces an accuracy, detection rate, and FAR of 97.66%, 94.88%, and 0.62 using the NSL-KDD dataset and MLP with 31 reduced features. Furthermore, Bharot et al. developed a novel IDS model for preventing cloud system attacks^41^. Before classifying network traffic into valid and malicious packets, the proposed model analyzes it using the Hellinger distance function. With a J48 classifier and the 12 most significant features from the NSL-KDD dataset, the system achieves detection rates of 99.81% and FARs of 0.12. In the same context, Chandak et al. developed a novel IDS to detect intrusions^42^. This model employs undersampling and oversampling approaches to reduce irregularities in the NSL-KDD dataset. The overall precision of the recommended detection approach is outstanding. However, this model lacks other evaluation criteria, including detection rate and accuracy.

Verma et al. suggest using an adaptive feature selection technique to identify cloud-based requests that are vulnerable to DDoS attacks^43^. Initially, the model computes the probabilities and entropies of the features. Second, the threshold values determined by dynamic threshold techniques include the interquartile range (IQR), mean absolute deviation (MAD), and median absolute deviation (MedAD). Finally, features are selected using threshold values. Compared to SVM, KNN, MLP, Adaboost, and DT, the MAD threshold technique produces the most accurate features, with a TPR of 98.226%. Likewise, Idhammad et al. presented an online sequential semi-supervised learning approach for detecting DDoS^44^. The performance of the proposed model is evaluated using the NSL-KDD, ISCX 12, and UNSW-NB15 datasets, achieving 98.23%, 93.71%, and 99.88% accuracy, respectively.

Recently, Soe et al. have proposed a lightweight IDS for the IoTs^45^. This approach combines the correlation coefficient and gain ratio for feature selection. Three distinct machine learning algorithms are used as analytic tools, including J48, RF, and DT. These algorithms are superior to the others in detecting DDoS and theft attacks. Similarly, Gao et al. propose an adaptive ensemble classification-based detection technique for intrusion detection using machine learning^46^. They use PCA to reduce the number of features in this model. The model’s accuracy was 85.2%. To deal with categorical data types, the authors of the studies used one-hot encoding, which increased the number of features from 42 to 122. In the same context, Hasan et al. suggested a machine learning-based anomaly detection solution for IoT sensors^47^. The suggested model performed better than the random forest. The DS2OS dataset is employed to evaluate this method. Moreover, Wu et al. developed a new IDS using convolutional neural network (CNN) technology to automatically find new features in a dataset^48^. This model’s rate of accuracy is 79.48%. Moreover, the author has applied one hot encoding to the training dataset, which has led to a reduction in the total number of features from 41 to 122. Kaja et al. recently suggested an intelligent IDS system using K-means clustering and various classifiers, including J48, RF, adaptive boosting, and NB^49^. They evaluated the proposed model using the KDD’CUP99 dataset, achieving an accuracy of 99.95%. In addition, Tian et al. proposed an IDS that employs an enhanced deep belief network^50^. According to the NSL-KDD and UNSW-NB15 datasets, the accuracy of the suggested model is 96.17 and 86.49%, respectively. With the help of deep belief networks (DBNs) and a modified density peak clustering method^51^, Yang et al. came up with a fuzzy aggregation method that looks for intrusions. The proposed model’s accuracy is 82.08%. Similarly, Pajouh et al. proposed a two-layer dimension reduction strategy and a two-tier classification method (TDTC) for anomaly-based IDSs. Moreover, they have used PCA and LCA to reduce the feature dimensions. In their two-tier approach to detecting malicious activity, they also use the factor versions of k-NN and NB. This model correctly identified 42% of U2R attacks and 78% of R2L attacks.

Software-Defined Networking (SDN) is an innovative model of network management that uses the control plane from the forwarding devices. This separation allows for centralized control of the network, enabling administrators to manage network behavior programmatically via software applications. Kumar et al.^52^ discussed the use of SDN to address the various challenges in detecting and mitigating DDoS attacks. They implemented several detection and mitigation techniques, along with defense strategies that leverage blockchain technology. Additionally, they explored the use of network slicing and honeypot strategies as defense mechanisms against DDoS attacks in SDN environments. The increasing adoption of IoT devices has introduced new security challenges because they often have limited processing power and memory. As a result, they can be vulnerable to various cyberattacks. As suggested in^24^, the authors have come up with a Hybrid Intrusion Detection System (HIDS) that combines the Quantum Support Vector Machine (QSVM) and the Improved Grey Wolf Optimizer (IGWO) algorithm to make IoT systems safer. They employed the Bot-IoT dataset for this purpose. Recently, Pakmehr et al.^53^ examined DDoS flood attacks to detect and defend mechanisms based on timing and location in IoT network systems. In addition, the authors investigated several solutions to identify and deal with DNS attacks.

**Table 1:** Presents a comprehensive summary of related works pertaining to strategies for cyber-attack detection in IoT networks.

References	Dataset	Machine learning algorithms	Deep learning algorithms	Metaheuristics algorithms	Evaluation criteria
14	UNBS-NB 15 KDD99	SVM, ANN, DT NB, K-means	-	-	Accuracy, FAR, Sensitivity, Specificity, FPR, AUC
15	NSL-KDD	-	Neural network	-	Accuracy
24	Bot-IoT	QSVM	-	IGWO	Accuracy, Recall, Precision, F1 score, ROC curve
39	NSL-KDD, AWID, CIC-IDS2017	Ensemble learning	-	CFS-BA	Accuracy
40	ISOT	-	MLP	-	Accuracy, Detection rate, FAR
41	NSL-KDD	J 48	-	-	Detection rate, FAR
42	NSL-KDD	ANN	-	-	Accuracy, precision Detection rate, recall, FAR
44	NSL-KDD, UNB ISCX 12, UNSW-NB15	ensemble learning	-	-	Accuracy, FPR
45	Bot-IoT	J48, RF, and DT	-	-	Accuracy, recall FPR, F1 score
46	NSL-KDD	DR, RF, KNN	-	-	Accuracy
47	DS2OS IoT Synthetic	LR, SVM, DT, RF, ANN	-	-	Accuracy, recall FPR, F1 score, AOC
48	NSL-KDD	-	CNN	-	Accuracy, FAR
49	KDD’CUP99	J48, RF, adaptive boosting, NB	-	-	Accuracy, FPR
50	NSL-KDD, UNSW-NB15	-	Improved deep belief network (DBN)	-	Accuracy, FPR
51	NSL-KDD, UNSW-NB15	-	Deep belief networks (DBNs)	-	Accuracy, recall, precision and F1-score
55	BoT-IoT	RF	CNN, MLP		Accuracy, AUC

## Preliminary

This section presented a brief introduction to the maximum correntropy criterion technique, the fuzzy C-means algorithm, and the sperm whale algorithm, along with some of the key concepts.

### Maximum correntropy criterion method

As discussed previously, feature selection techniques are essential for detecting IoT cyberattacks. By minimizing the number of initial features, feature selection reduces model complexity, overfitting, model computing efficiency, and generalization error. We compute the similarity of the suggested feature vectors using the maximum correntropy criterion (MCC), which accurately distinguishes between normal and attack instances. We used MCC to measure the similarity of the suggested feature vectors, and it accurately distinguishes between normal and attack occurrences.

Consider the desired vector of features $\:{m}_{i}$ that describes the most relevant features of data set and generates from identification model $\:{m}_{k}={u}_{k}^{T}{w}_{o}+{e}_{k},$ where $\:{w}_{o}$ represents an estimated coefficient weight vector, $\:{u}_{i}$ represents the input vector $\:{\varvec{u}}_{\varvec{k}}\:=\:{({u}_{k},{u}_{k-1},\dots\:,{u}_{k-L+1})}^{T}$ and $\:{e}_{k}$ is the error rate. The error rate is calculated as $\:{e}_{k}={m}_{k}-{u}_{k}^{T}{w}_{k},\:$ where $\:{w}_{i}$ is the calculated $\:{w}_{o}$ at iteration $\:k$. The Mean Square Error (MSE) is employed here to compare between any two feature vectors. It is used to compare any two feature vectors $\:X\:and\:\stackrel{\sim}{X}$ as follows:

1 $$\:{M}_{global}\left(X,\stackrel{\sim}{X}\right)=E\left[{\left(x-\stackrel{\sim}{x}\right)}^{2}\right]$$

The cost function of the MSE represented as follows:

2 $$\:{V}_{MSE}\left({w}_{k}\right)=E\left[{e}_{k}^{2}\right]$$

By utilizing a stochastic gradient ascent method, we can get

3 $$\:{w}_{k+1}={w}_{k}+\mu\:\nabla\:{\varvec{u}}_{\varvec{i}}{e}_{k}$$

Where $\:\mu\:$ and $\:\nabla\:\:$respectively represent the step-size and gradient operator. Here, the proposed MCC provides a more robust solution for impulsive interference. For any two discrete vectors $\:X\:and\:\stackrel{\sim}{X}$, the correntropy measure is defined as follow:

4 $$\:{M}_{\sigma\:}\left(X,\stackrel{\sim}{X}\right)=E\left[{k}_{\sigma\:}\left(x-\stackrel{\sim}{x}\right)\right]$$

Where $$\:{\text{K}}_{{\upsigma\:}}$$ is the Gaussian kernel function that satisfying the Mercer theory and without loss of generality, the Gaussian kernel given as $$\:{M}_{\sigma\:}\left(X,\stackrel{\sim}{X}\right)=\frac{1}{\sqrt{2\pi\:\sigma\:}}\text{exp}\left(-\left(\raisebox{1ex}{${e}^{2}$}\!\left/\:\!\raisebox{-1ex}{$2{\sigma\:}^{2}$}\right.\right)\right)$$ Where $$\:e=\:x-\stackrel{\sim}{x}\:$$, and $$\:\sigma\:$$ denotes the kernel bandwidth and requires$$\:\:\sigma\:>o$$. By using the Taylor series expansion to (2), we have

5 $$\:{M}_{\sigma\:}\left(X,\stackrel{\sim}{X}\right)=\:\frac{1}{\sqrt{2\pi\:\sigma\:}}\:\sum\:_{r=1}^{\infty\:}\frac{{\left(-1\right)}^{r}}{{2}^{r}r!}\:E\left[\frac{{e}^{2r}}{{\sigma\:}^{2r}}\right]$$

Where the error $\:e$ is defined as $\:e=x-\stackrel{\sim}{x}$. Specifically, the maximum correntropy of the error vector $\:e={\left({e}_{1},{e}_{2},\dots\:,{e}_{n}\right)}^{T}$ is defined as follows:

6 $$\:\underset{\theta\:}{\text{max}}\:\frac{1}{n}\sum\:_{i=1}^{n}{M}_{\sigma\:}\left({e}_{i}\right)$$

Where $\:\theta\:$ is the parameter in the criterion to be optimized.

### The fuzzy C-means algorithm

**Fig. 2:**
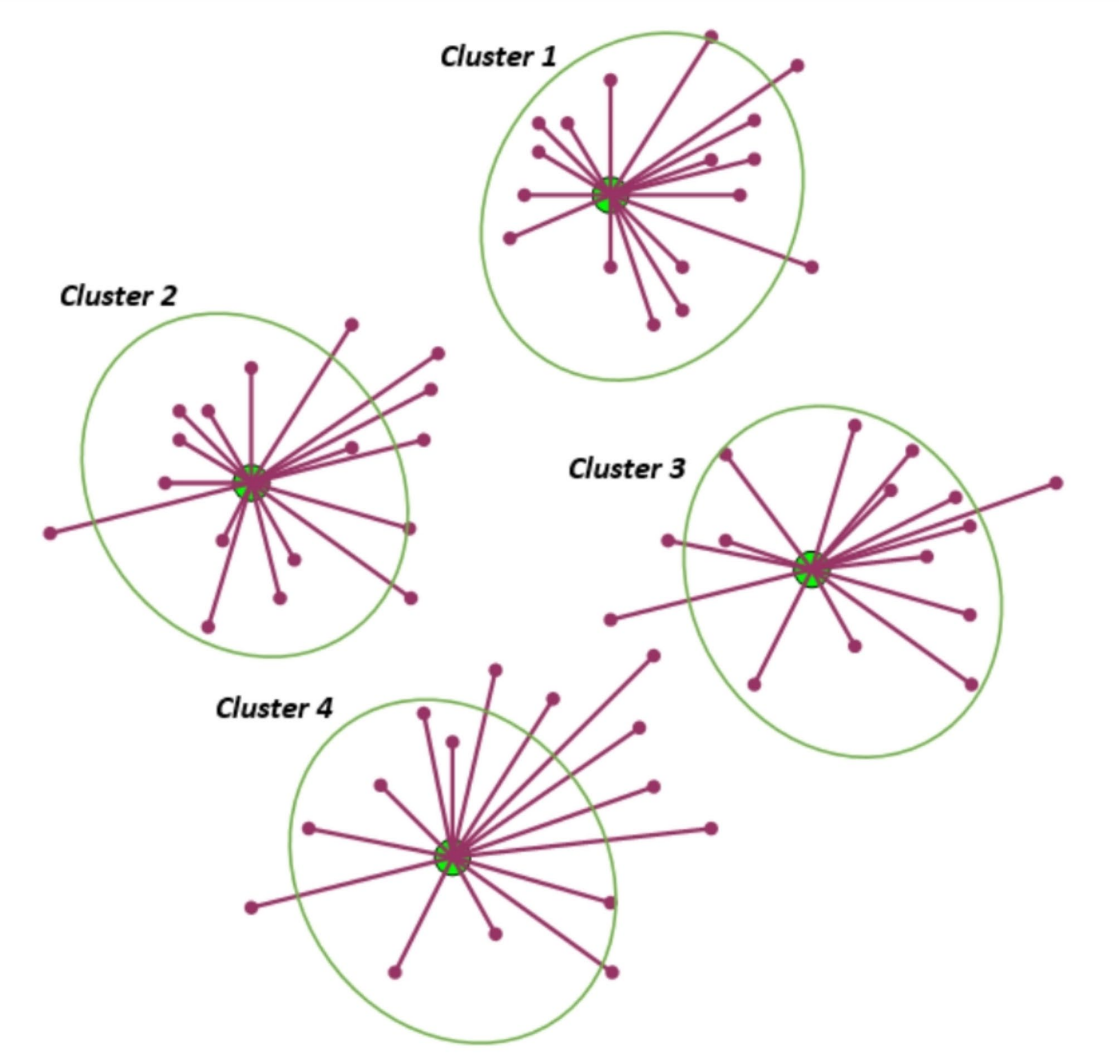
The fuzzy C-means clusters.

The fuzzy C-means algorithm (FCM)is one of the most often used strategies for attack detection in this setting because of its improved overall performance compared to other ML algorithms^29–33^. It is one of the most widely used semi-supervised learning techniques that trains on both labeled and unlabeled data to extract the best features from the target data set. As demonstrated in Fig. 2, FCM reduces the non-similarity index value, divides n vectors into c fuzzy groups, and identifies the clustering centers of each group. Fuzzy partitioning is used to classify each piece of information, converting each value into a number between 0 and 1. Conceptually, FCM enhances clustering by allowing a data point to be allocated to numerous classes with varied degrees of membership.

The process of FCM algorithm begins considering that $\:R=\left\{{r}_{1},{r}_{2},\dots\:,\:{r}_{n}\:\right\}$ denotes a data set consisting of *n* samples in *d*-dimensional space, and $\:V=\left\{{v}_{1},{v}_{2},\dots\:,\:{v}_{m}\:\right\}$ is the set of *m* centroids. To classify samples, the algorithm employs an iterative process in which an objective function defined as follows is minimized.

7 $$\:\text{min}{F}_{p}=\:\sum\:_{i=1}^{m}\sum\:_{j=1}^{n}{{{\left({u}_{ij}\right)}^{p}\left|\right|r}_{\text{j}}-{v}_{\text{i}}\left|\right|}^{2}$$

Where $\:1\le\:p\le\:\infty\:$ represents the *fuzzifier*. The degree of membership for data set $\:R$ with *n* samples to *m* centroids can be defined as partition matrix $\:T=\:{\left\{{u}_{ij}\right\}}_{mxn}$, and $\:p$ is the fuzziness degree of matrix $\:T$. $\:{u}_{ij}\in\:\left[\text{0,1}\right]$ represents the fuzzy membership and satisfies two conditions: $\:\sum\:_{i}^{m}{u}_{ij}=1$ and $\:0{\le\:u}_{ij}\le\:1$. $\:{{d}_{ij=}\left|\right|r}_{\text{j}}-{v}_{\text{i}}\left|\right|$ is the distance between sample $\:{r}_{j}$ and cluster center $\:{v}_{\text{i}}$. When the target samples are near the cluster center, they are given high membership values. On the other hand, when they are far from the centroid, they award low membership values. Further, the method membership functions $\:{u}_{ij}$ and cluster centers $\:{v}_{\text{i}}$ are updated using the Eqs. (8) and (9) respectively.

8 $$\:{u}_{ij}=\:\frac{1}{{\sum\:}_{k=1}^{n}{\left(\frac{{d}_{ij}}{{d}_{ki}}\right)}^{\frac{2}{p-1}}}\:\:\:,\:where\:\:{{d}_{ij}=\left|\right|r}_{\text{j}}-{v}_{\text{i}}\left|\right|$$

9 $$\:{v}_{\text{i}}=\:\frac{1}{{m}_{i}}\sum\:_{i=1}^{m}{\left({u}_{ij}\right)}^{p}\:{r}_{i}\:\:\:\:,\:where\:\:\:{m}_{i}=\sum\:_{i=1}^{m}{\left({u}_{ij}\right)}^{p}$$

### Sperm whale algorithm

**Fig. 3:**
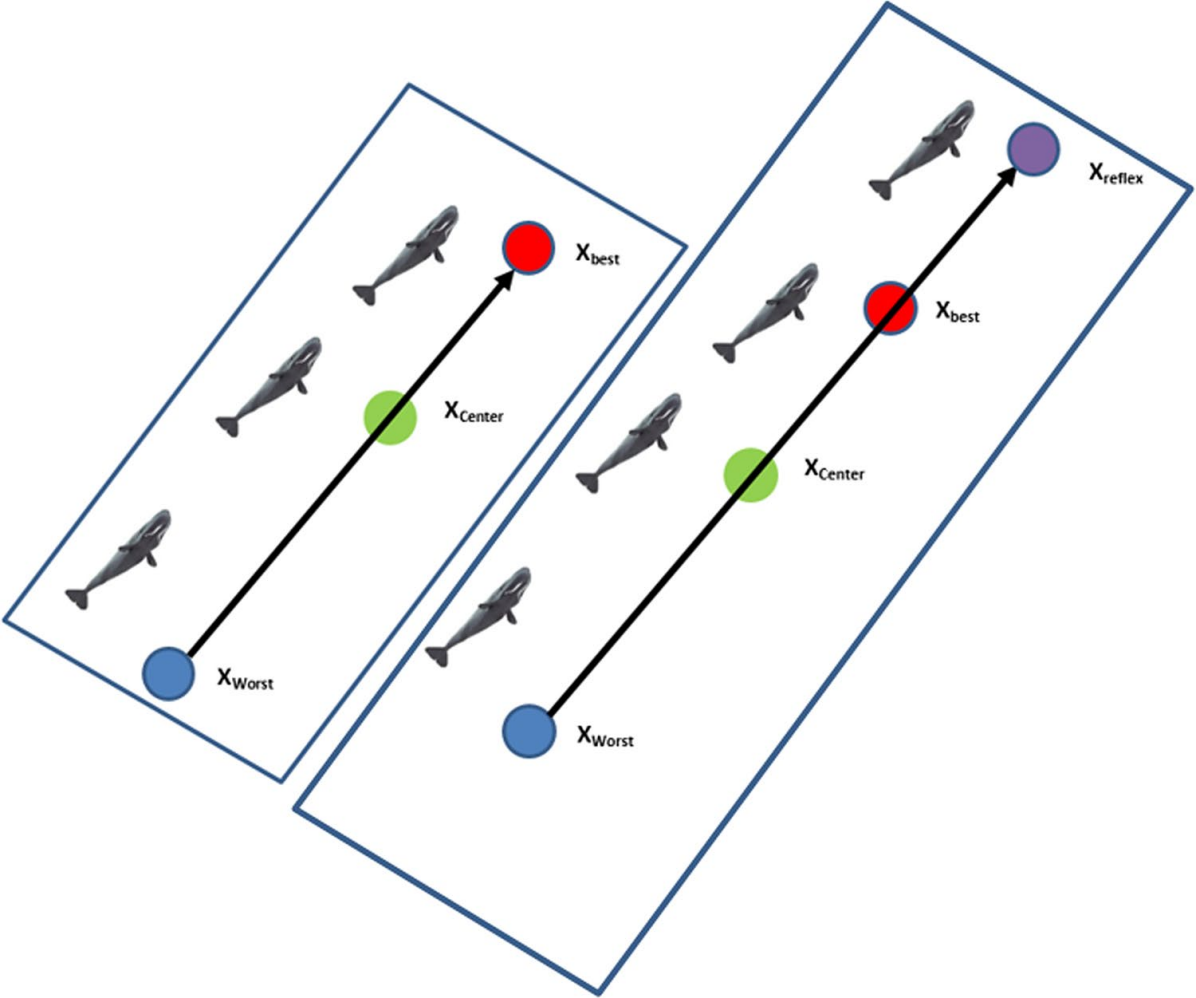
Exploration and Exploitation of SWA in the search space.

The Sperm Whale Algorithm (SWA) is a novel metaheuristic method presented by Ebrahimi and Khamehci^38^. The Sperm whale’s lifestyle served as its inspiration. In SWA, an initial population of *mx n* individuals should be generated. After that, the primary population is divided into *n* temporary subgroups (TSG) containing m individuals. Then, a randomly selected TSG generates a new main sub-group (MSG) with *n* members. The purpose of creating several subgroups is to prevent SWA from terminating prematurely and stuck in local minima. As shown in Fig. 3 the positions of the leader and the worst member of each group, *X*_*best*_ and *X*_*worst*_, are utilized to calculate an intermediate distance, *X*_*dist*_. Because each sperm whale travels through two places throughout its breathing–feeding cycle, the cost function will be constructed using both positions (current position and relative position).

**The main process of the SWA is presented in Algorithm 1 and its main steps can be described as follows**:

**Step1**: The primary population of sperm whale is initialized randomly, with assigning matrix.

$\:{W}_{mxn}=({w}_{11},{w}_{11},\dots\:,{w}_{1n},{w}_{21},\dots\:,{w}_{2n},\dots\:,{w}_{mn})$. Where the initial population is divided into $\:n$ Temporary Sub-group (TSG), each containing $\:m$ members.

**Step2**: Each sperm whale is evaluated according to the quality of its two positions (i.e., breathing position at the surface and feed position at seabed).

**Step3**: Sperm whale creates a new location according to the best and worst whale in each group taking the information exchange between whales into account. Assume that at iteration *t* the leader and worst sperm whales in group *g* are named as, and, respectively, so.

10 $$\:\:{dist}^{(g,t)}=\:\:{worst}^{(g,t)}+\rho\:.\:{leader}^{(g,t)}$$

11 $$\:\:{reflex}^{(g,t)}=\:\:{worst}^{(g,t)}+2.(\:{dist}^{(g,t)}\:-\:{worst}^{(g,t)})\:=\:2.\:{dist}^{(g,t)}-{worst}^{(g,t)}$$

Where $\:{dist}^{(g,t)}$ is denoted as the reflection center and $\:\rho\:\:$refers to center factor. Besides, $\:{reflex}^{(g,t)}$ is the result obtained from $\:{worst}^{(g,t)}$ to $\:{dist}^{(g,t)}$.

**Step4**: When $\:{reflex}^{(g,t)}$ is outside the search space. In this case, $\:\rho\:\:$should defined in this way: $\:\:\rho\:=r\:\times\:\:{\rho\:}_{i}$, in which $\:{\rho\:}_{i}$ is the primary central factor and $\:r$ named the shrinkage factor is less than 1. Furthermore, $\:H$ is presumed as a *1×n* vector and *n* is the number of decision variables. Then,

$$\:for\:i=1\::\:n$$

$$\:{min}_{i}^{(g,t)}\le\:{reflex}_{i}^{(g,t)}\le\:{max}_{i}^{(g,t)}$$

$$\:{min}_{i}^{(g,t)}\le\:2.{dist}_{i}^{(g,t)}-{worst}_{i}^{(g,t)}\le\:{max}_{i}^{(g,t)}$$

$$\:{min}_{i}^{(g,t)}\le\:2\times\:\left({worst}_{i}^{(g,t)}+{H}_{i\:}\times\:{leader}_{i}^{(g,t)}\right)-{worst}_{i}^{(g,t)}\le\:{max}_{i}^{(g,t)}$$

$$\:min_{i}^{{(g,t)}} \le \:\left( {worst_{i}^{{(g,t)}} + 2{\text{~}}H_{{i\:}} \times \:leader_{i}^{{(g,t)}} } \right) \le \:max_{i}^{{(g,t)}}$$

$$\:\frac{{min}_{i}^{(g,t)}-{worst}_{i}^{(g,t)}}{2.{leader}_{i}^{(g,t)}}\le\:{H}_{i\:}\le\:\frac{{max}_{i}^{(g,t)}-{worst}_{i}^{(g,t)}}{2.{leader}_{i}^{(g,t)}}$$

Then, from each Main Sub-Group (MSG), a certain number of whales (*k*) with higher fitness scores are chosen to create a group known as the “Good Gang.“.

**Step5**: After finding the optimal solution for the Good Gang group, we can observe the crossover with other MSG groups.

**Step6**: Finally, when the maximum number of iterations is reached, SWA terminates and returns the best response vector, which is the optimal solution set for the optimization problem. If the maximum number of iterations is not reached, the SWA will run until the end of the loop.

**Figure:**
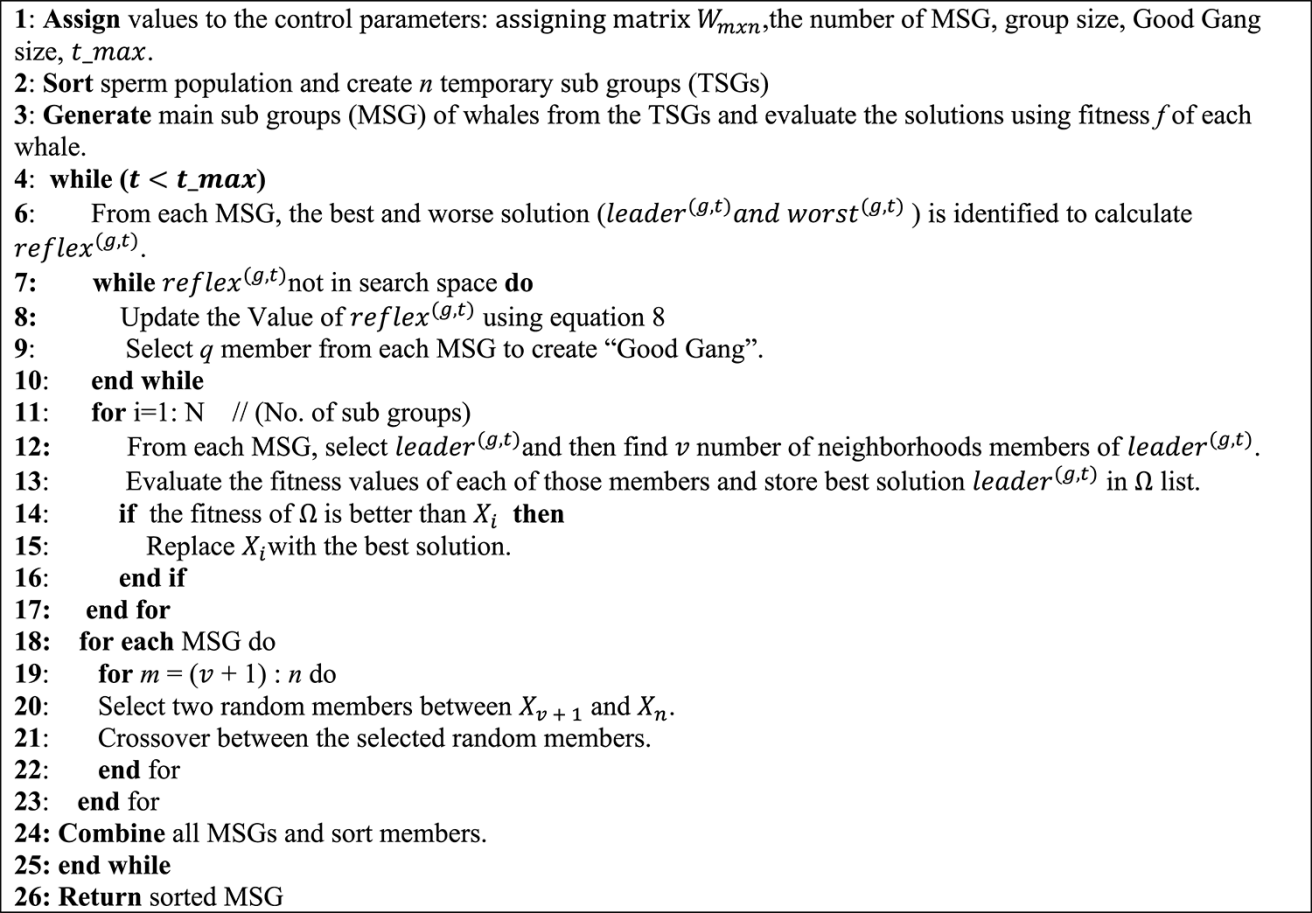
Algorithm 1 Sperm whale algorithm (SWA)

### The proposed FCM-SWA approach for cyber-attack detection in IoT networks

**Fig. 4:**
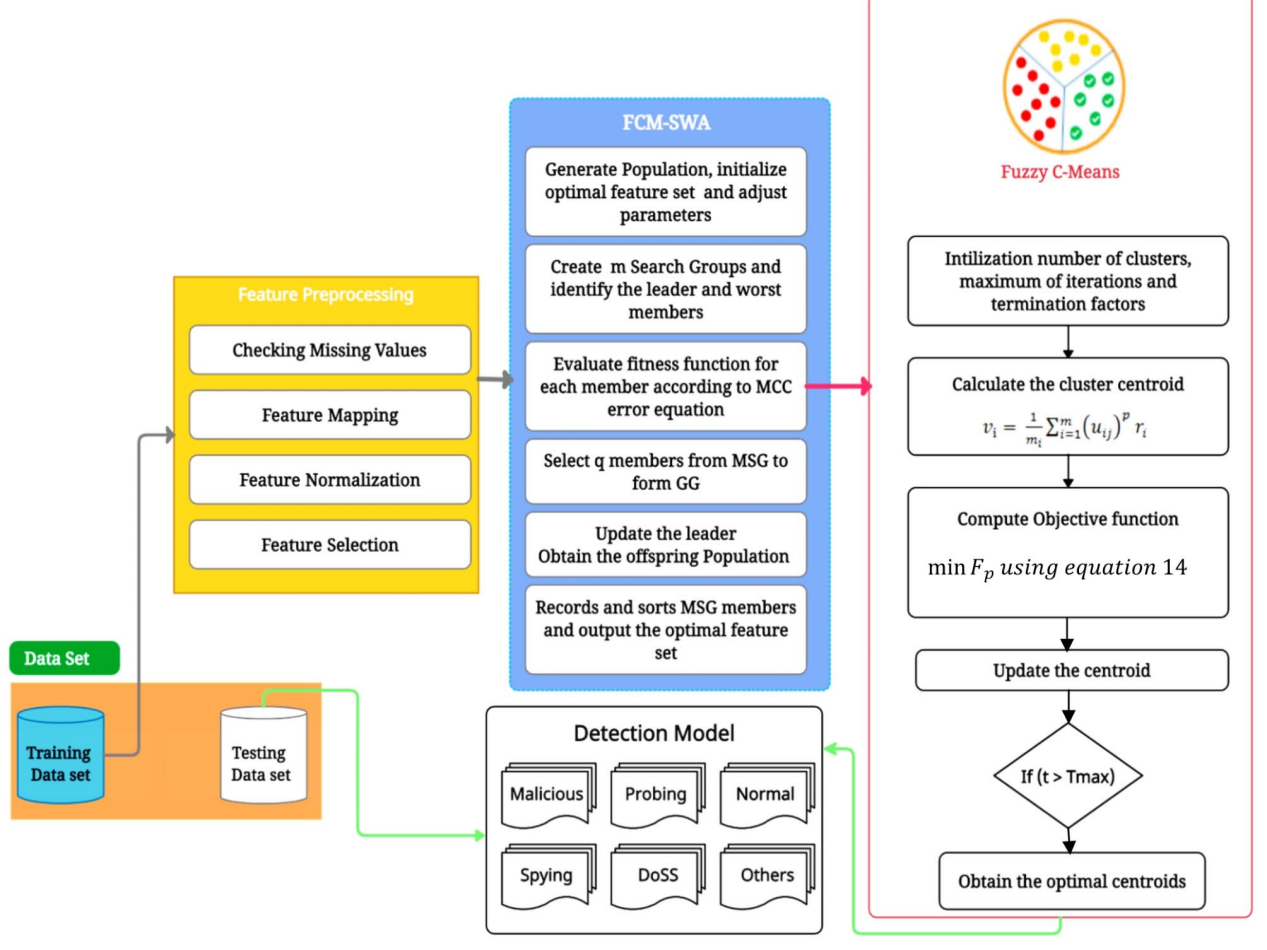
The proposed FCM-SWA model serves as a pipeline for detecting IoT cyber-attacks, utilizing fuzzy C-means and the Sperm Whales Algorithm.

This section describes an intelligent cyber-attack detection model, FCM-SWA, that efficiently detects botnets in IoT networks for smart cities. Figure 4 illustrates the proposed FCM-SWA model, which uses fuzzy c-means and the Sperm Whales algorithm to detect IoT cyberattacks. The initial steps involve the preprocessing of incoming IoT network data, feature mapping, and feature normalization. The subsequent phase uses the MCC approach to rank and select features based on the data. The final step utilizes a hybrid FCM-SWA algorithm for feature selection, identification, and decision-making. The following sections provide a detailed description of the processes and features of each phase.

- **Features Preprocessing**.

This section provides an in-depth overview of the feature preprocessing approaches utilized by the proposed detection model. We preprocess the raw data to eliminate noise and missing data once we collect the incoming IoT network traffic. In an IoT-enabled smart environment, a number of devices and sensors generate traffic with a variety of characteristics, including quantitative and qualitative data. Data cleaning, normalization, transformation, and integration are the four types of available preprocessing procedures. Data cleaning is the elimination and correction of inaccurate, duplicate, corrupted, and erroneous data from datasets. The primary objective is to clean the dataset in order to standardize data analysis and make it easier to discover the appropriate data for a query.

- **Feature Mapping**.

A large collection of IoT network traffic may contain a variety of category variables. Numerous machine learning techniques do not support these variables. The one-hot encoder technique can be utilized as a workaround; however, this will gradually increase the number of features and make the dataset sparse. The sparse characteristics of a dataset make it more difficult to fit it into the suggested FCM-SWA model and increase processing time. Here, label-one-hot encoding assigns a unique number to each subclass. This stops the dataset from becoming sparse and restricts the feature’s growth.

- **Feature Normalization**.

Normalization is the process of rescaling real-valued numeric features to the 0 to 1 interval. Normalization enhances consistency in the training data, enabling the model to predict outputs more accurately. We use the Euclidean distance to determine the closeness of the features. For any two feature vectors $\:x=\left({x}_{1},{x}_{2},\dots\:,{x}_{n}\right)$ and $\:y=\left({y}_{1},{y}_{2},\dots\:,{y}_{n}\right)$ in 2-dimensional space, the theorem states that the Euclidean distance between them may be computed using Eq. (12). However, if features with large magnitude values are present, all distance-based classifiers will perform poorly due to their increased weighting. The suggested detection method uses the min-max normalization strategy to evaluate features and improve convergence and training time, as illustrated below.

12 $$\:\varvec{d}\varvec{i}\varvec{s}\varvec{t}\left(\varvec{x},\varvec{y}\right)=\sqrt{\sum\:_{\varvec{i}=1}^{\varvec{n}}{\left({\varvec{x}}_{\varvec{i}\:-}{\varvec{y}}_{\varvec{i}}\right)}^{2}}$$

13 $$\:{\varvec{X}}_{\varvec{n}\varvec{e}\varvec{w}}=\frac{\left({\varvec{X}\:\--\varvec{X}}_{\varvec{m}\varvec{i}\varvec{n}}\right)}{\left({\varvec{X}}_{\varvec{m}\varvec{a}\varvec{x}}\:\--{\:\varvec{X}}_{\varvec{m}\varvec{i}\varvec{n}}\right)}$$

Where $\:\varvec{X}$ is the feature to be scaled down, $\:{\varvec{X}}_{\varvec{m}\varvec{a}\varvec{x}}$ is the highest value, and $\:{\:\varvec{X}}_{\varvec{m}\varvec{i}\varvec{n}}$is the minimum value for a certain feature in the dataset.

- **Fitness function**.

The traditional FCS algorithm is sensitive to the initial cluster centers, labeled data, and unlabeled data. The main problems of FCM are slow convergence, sticking to local optimal solutions, and poor stability. We integrate FCM with the Sperm Whale Algorithm, known as FCM-SWA, to overcome these problems and increase its accuracy. The FCM-SWA employs the sperm whale algorithm to resolve the clustering problem and identify the optimal cluster center, thereby achieving a balance between exploration and exploration in the solution search space. We propose a new distance measure based on the degree of membership of labeled and unlabeled data. Formally, let $\:R=\left\{{r}_{1},{r}_{2},\dots\:,\:{r}_{n}\:\right\}$ denotes unlabeled dataset and $\:\stackrel{\prime }{R}=\left\{\stackrel{\prime }{{r}_{1}},\stackrel{\prime }{{r}_{2}},\dots\:,\:\stackrel{\prime }{{r}_{n}}\:\right\}$, where $\:\stackrel{\prime }{{r}_{j}}\in\:{\text{R}}^{\text{d}}$ is labeled dataset of dimension *d* and size *n*. Here, FCM-SWA approach utilizes membership functions to form the fitness functions as follows.

14 $$\:\text{min}{F}_{p}=\:\sum\:_{i=1}^{m}\sum\:_{j=1}^{n}{{{\left({u}_{ij}\right)}^{p}\left|\right|r}_{\text{j}}-{v}_{\text{i}}\left|\right|}^{2}\:+\:\sum\:_{i=1}^{m}\sum\:_{j=1}^{\stackrel{\prime }{n}}{{{\left({\stackrel{\prime }{u}}_{ij}\right)}^{p}\left|\right|\stackrel{\prime }{r}}_{\text{j}}-{v}_{\text{i}}\left|\right|}^{2}$$

Where for labeled data point $\:\stackrel{\prime }{{r}_{\text{i}}}$ the membership functions $\:{\stackrel{\prime }{u}}_{ij}$ is defined as:

15 $$\:{\stackrel{\prime }{u}}_{ij}=\:\frac{1}{{\sum\:}_{k=1}^{n}{\left(\frac{{\stackrel{\prime }{d}}_{ij}}{{\stackrel{\prime }{d}}_{kj}}\right)}^{\frac{2}{p-1}}}$$

In Eqs. (14) and (15), $\:{\stackrel{\prime }{u}}_{ij}$ is the degree of membership for labeled data, satisfying two conditions: $\:\sum\:_{i}^{m}{\stackrel{\prime }{u}}_{ij}=1$ and $\:0\le\:{\stackrel{\prime }{u}}_{ij}\le\:1$. $\:{{\stackrel{\prime }{d}}_{ij}=\left|\right|r}_{\text{j}}-{v}_{\text{i}}\left|\right|$ is the distance between sample $\:\stackrel{\prime }{{r}_{j}}$ and cluster center $\:{v}_{\text{i}}$.

The FCM-SWA approach’s entire process is depicted in Algorithm 2 and is detailed as follows:

**Figure:**
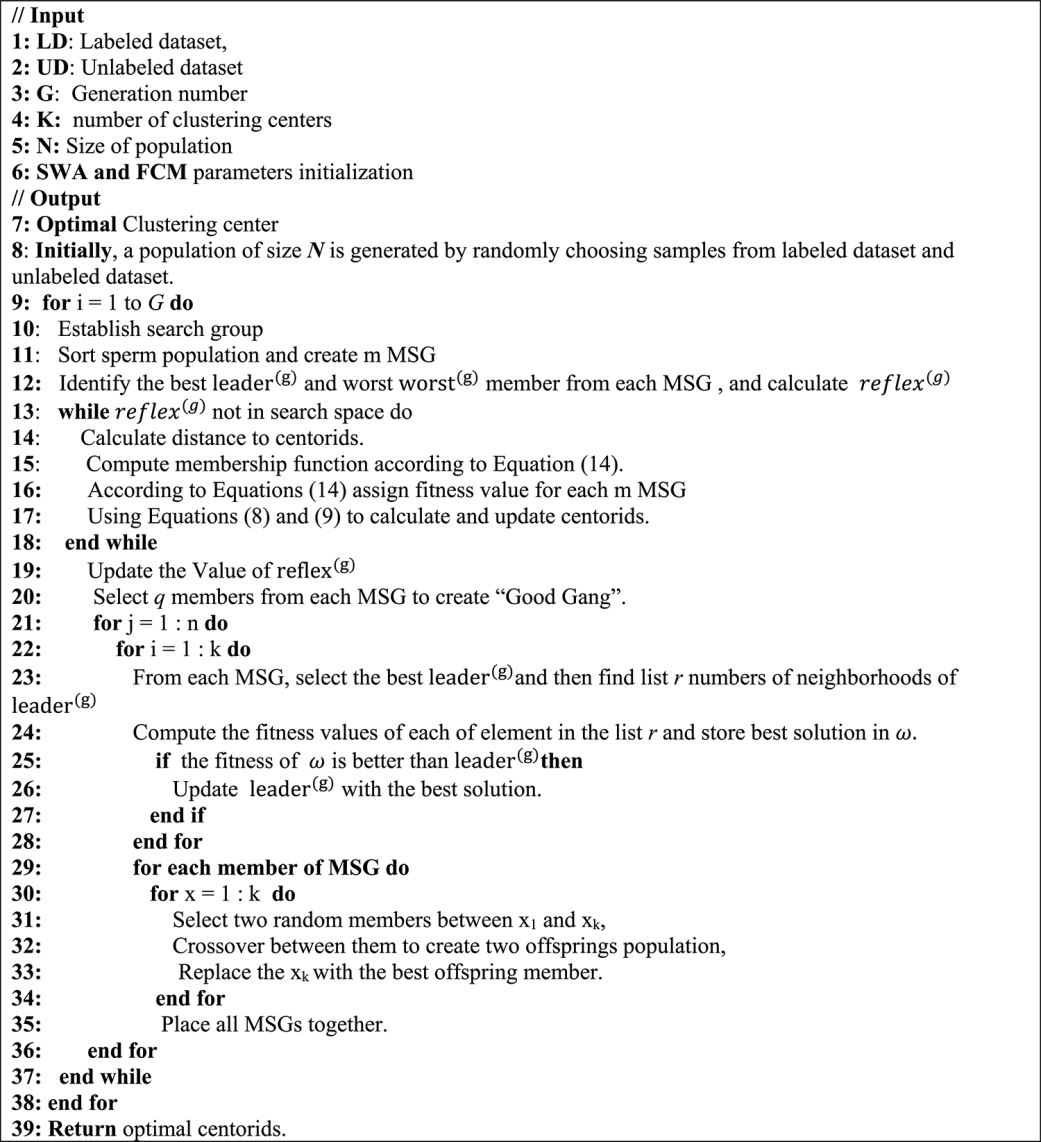
Algorithm 2 The proposed algorithm for FCM-SWA approach

The FCM-SWA hybrid technique combines the clustering power of FCM with the optimization power of SWA to make a strong framework for finding cyberattacks in IoT networks. This hybrid method enhances detection accuracy and scalability, making it appropriate for the complex and dynamic nature of IoT environments. It satisfies the criteria for real-time and efficient detection systems by adeptly grouping ambiguous network data and improving the classification process to distinguish between normal and malicious activities. Initially, we employ FCM to categorize the network traffic data into clusters. These clusters denote several sorts of traffic, including typical activities or possible threats. However, FCM alone may encounter challenges in effectively identifying the cluster centers. We then use SWA to refine the cluster centers that FCM has identified. This guarantees that the clusters are more accurate, efficiently distinguishing regular network traffic from unusual behavior that may signify a cyberattack. SWA, a robust meta-heuristic optimization method, tackles complex problems like intrusion detection in large-scale IoT networks. In cyber-attack detection, the SWA enhances the clustering process by directing the FCM toward more precise clusters and ensuring optimal differentiation between normal traffic and dangerous attacks. SWA emphasizes the equilibrium between exploration (investigating new areas within the solution space) and exploitation (concentrating on promising locations). It employs position-update techniques informed by a fitness function, directing solutions toward the optimal location. The integration of FCM’s fuzzy clustering with SWA’s optimization enhances the precision of cyber-attack detection, minimizing both false positives and false negatives. The SWA guarantees rapid convergence of the clustering process to an ideal solution, essential for real-time IoT networks handling substantial data volumes. Scalability: The hybrid methodology is engineered to accommodate the volume and intricacy of IoT ecosystems where network configurations and data may fluctuate swiftly.

The FCM-SWA hybrid approach is complicated because it combines FCM clustering SWA. It’s important to look at the computational costs of both FCM and SWA separately, as well as how they work together in the hybrid approach. The time complexity of calculating distances is O (*I*_*FCM*_.*N*⋅*C*⋅*F*) for a single iteration. The variables N, C, and F represent the number of iterations until convergence, the number of data points, the number of clusters, and the number of features per data point, respectively. The SWA is a nature-inspired optimization algorithm, and its complexity stems from the iterative search and optimization process. The exploration and exploitation phases, where SWA adjusts the positions of candidate solutions, incur the primary computational cost. The overall time complexity of SWA is O(*I*_*SWA*_⋅*W*⋅*D*), where ISWA, W, and D are the number of iterations until convergence, number of dimensions, and number of candidate solutions, respectively. The total time complexity of the FCM-SWA hybrid approach is the sum of the complexities of FCM and SWA as O (*I*_*FCM*_.*N*⋅*C*⋅*F*) + O (*I*_*SWA*_⋅*W*⋅*D*).

## Experimental results and discussion

This section evaluates and compares the performance of the proposed FSM-SWA model to previously developed IDS and related state-of-the-art techniques.

### Dataset and computer system description

The experiment was conducted on a Google Colab instance with a Xeon processor at dual 2.20 GHz and 16 GB of RAM, and the suggested FCM-SWA detection model was developed using Python 3. Obtaining a sufficient dataset is one of the challenges faced by researchers when analyzing the proposed model. Thus, we evaluated the performance of the proposed FCM-SWA model using three datasets: NSL-KDD, Aegean WiFi Intrusion Dataset (AWID), and BoT-IoT. Table 2 depicts the statistics of samples from various classes within the target datasets.

- **Aegean WiFi intrusion dataset**.

The Aegean WiFi intrusion dataset (AWID) is a collection of WiFi network datasets, which include real records of both regular and intrusive data collected in real-world networks^33^. The dataset uses a total of 155 features, either numerical or nominal, to represent each record, and Table 2 provides additional details on the number of individual attacks. We divide the dataset into four groups: (I) normal, (II) impersonation, (III) injection, and (IV) flooding. The fourth category, “normal traffic,” represents secure connections, while the first three categories reflect intrusion attempts.

- **NSL-KDD dataset**.

The NSL-KDD dataset was released in 2009 as an expansion of the KDDCup’99 dataset^32^. By eliminating duplicates, normalizing the number of instances, and maintaining the diversity of chosen samples, NSL-KDD overcame several shortcomings of the KDDCup’99 data set. NSL-KDD includes a total of 41 features plus one target class. We can distinguish four categories of network attacks based on the value of the variable “target”. Table 2 provides information about the attack datasets used in this study. The considered benchmark dataset contains a total of 119,241 samples, which are divided into four categories: normal, DoS, user-to-root attack (U2R), and probe attack. Table 2 shows the frequency of various types of attacks and anomalies in the whole dataset. We discovered the following categories with the NSL-KDD dataset.

(i) **Normal**: In this type, authorized users first attempt to modify their account’s privileges before resuming normal operations.
(ii) **Denial of Service (DoS)**: During a denial-of-service attack, the attacker will repeatedly send bogus messages over the network and demand that the server verifies their authenticity using a fake return address. This technique causes the server to become overcrowded, making it impossible for legitimate users to access the service.
(iii) **User-to-Root attack (U2R)**: After gaining access as a regular user, the attacker makes a first effort to elevate their privileges to those of the root user. A root user has complete control over all system resources on a network.
(iv) **Probe attack**: These attacks aim to search a network for vulnerabilities that they can later exploit.

- **BoT-IoT dataset**.

The UNSW Canberra Cyber Range Centre gathered the BoT-IoT dataset^34^ in 2018 through the construction of real-world network infrastructure. The MQTT protocol, for message queuing telemetry transfer, generates this data. The dataset provides summaries of the various forms of attacks.

(i) **DDoS attacks**: The term “distributed denial-of-service” (DDoS) refers to an attack in which many targets are simultaneously overwhelmed by traffic from a large number of IoT devices. DDoS attacks are getting worse as the number of connected IoT devices grows.
(ii) **DoS attacks**: A denial of service attack floods traffic or hosts until they crash or become unresponsive, preventing legitimate IoT devices from connecting.
(iii) **Reconnaissance attacks**: Reconnaissance is the process of acquiring or testing information in order to determine a network’s vulnerabilities, which are subsequently used to begin a successful attack. Inspection of traffic and packet sniffers are instances of reconnaissance.
(iv) **Theft attacks** are a set of attacks designed to breach the defenses of the IoT system in order to obtain private information. Common forms of theft include data theft and keylogging.

### Measure for performance evaluation

In this section, we present an extensive variety of performance matrices used to evaluate the proposed model, including accuracy, precision, sensitivity, F-score, area under the receiver operating characteristic curve, true positives, true negatives, false positives, and false negatives. Each of these indicators offers a valuable perspective on the model’s performance and has the following definition:

- **Accuracy (AC**) is measured as the proportion of properly classified samples to the sum of all predictions:

16 $$\:\varvec{A}\varvec{C}=\frac{\varvec{T}\varvec{P}+\varvec{T}\varvec{N}}{\varvec{T}\varvec{P}+\varvec{T}\varvec{N}+\varvec{F}\varvec{P}+\varvec{F}\varvec{N}}\times\:100$$

- **Detection rate (DR)**, also known as sensitivity or true positive rate, is the proportion of identified positives within a dataset. It is also called “Recall” and has the following definition:

17 $$\:\mathbf{D}\mathbf{R}=\frac{\varvec{T}\varvec{P}}{\varvec{T}\varvec{P}+\varvec{F}\varvec{N}}\times\:100$$

- **Precision rate(PR)**: It indicates the ratio of truly classified positives to the total actual number of predicted positives made by the proposed model and can be defined as follows:

18 $$\:\mathbf{P}\mathbf{R}\:=\frac{\varvec{T}\varvec{P}}{\varvec{T}\varvec{P}+\varvec{F}\varvec{P}}\times\:100$$

- **F-Score**: It computes an aggregate measure of accuracy based on recall and precision. rate. It is more valuable than accuracy since it determines the false-positive (FP) and false-negative (FN) rates and is used mostly when the class distribution is uneven (FN). It is calculated as follows:

19 $$\:\mathbf{F}1\:\mathbf{S}\mathbf{c}\mathbf{o}\mathbf{r}\mathbf{e}\:=2\times\:\frac{\mathbf{P}\mathbf{r}\mathbf{e}\mathbf{c}\mathbf{i}\mathbf{s}\mathbf{i}\mathbf{o}\mathbf{n}\:\times\:\mathbf{R}\mathbf{e}\mathbf{c}\mathbf{a}\mathbf{l}\mathbf{l}}{\mathbf{R}\mathbf{e}\mathbf{c}\mathbf{i}\mathbf{s}\mathbf{i}\mathbf{o}\mathbf{n}+\:\mathbf{R}\mathbf{e}\mathbf{c}\mathbf{a}\mathbf{l}\mathbf{l}}\times\:100$$

- **AUC** is an abbreviation for “area under the receiver operating characteristic” curve. A common metric to demonstrate a detection model’s ability to distinguish between benign and malicious attacks is the area under the receiver operating characteristic (ROC) curve, which also performs well with data sets that exhibit class imbalance. Each point on the ROC graph represents the sensitivity/specificity at a certain cutoff value. An AUC with a higher value indicates superior performance. Here is a simple definition for the AUC.

20 $$\:\varvec{A}\varvec{U}\varvec{C}=\:{\int\:}_{\varvec{a}}^{\varvec{b}}\varvec{f}\left(\varvec{x}\right)\varvec{d}\varvec{x}$$

Where, to measure the AUC value, *y = f(x)* among the x = a, and x = b with an integral of *y = f(x)* within the limit of a and b.

**Table 2:** A description of the benchmark datasets used for training and testing based on the proposed FCM-SWA.

Samples distribution for AWID dataset				
	**Normal**	**Flooding**	**Impersonation**	**Injection**
Training	1,633,190	48,484	48,522	65,379
Testing	326,638	9,696	9,704	13,075
Total	1,959,828	58,180	58,226	78,454
Samples Distribution for NSL-KDD dataset				
	Normal	DoS_attacks	Probe_attacks	U2R
Training	67,343	45,927	11,656	995
Testing	13,468	9,185	2,331	199
Total	80,811	55,112	13,987	1,194
Samples Distribution for BoT-IoT dataset				
	DDoS attacks	DoS attacks	Reconnaissance	Theft attacks
Training	1,348,636	1,137,181	72,919	65
Testing	269,727	227,436	14,583	13
Total	1,618,363	1,364,617	87,502	78

### Detection performance

The performance of the proposed IDS model, FCM-SWA, is measured by its ability to correctly identify IoT traffic. As indicated in Table 3, the label encoding is applied to the various classes seen in the target datasets, which merely replaces each label with a unique number. Then, this transformation is done to the validation and testing subsets. The first experiment is carried out to determine the effectiveness of the proposed FCM-SWA with respect to the size of the dataset. Here, 80% of each dataset is used for training, while the remaining 20% is used for testing. Table 4 indicates that as the size of the dataset increases, TP increases while FP decreases, which is consistent with expectations. This is because when the dataset size is too small, the evolution technique may be unable to adequately narrow the solution space, resulting in a decrease in average searching quality. Table 5 shows the confusion matrix for 20,000 DoS attack events from the BoT-IoT data collection that was utilized for training. The model can detect attacks on smart city networks with 97% precision by minimizing both FP and FN. Since the FN indictor is a threat to IoT networks that must be identified and addressed, it is obvious that FCM-SWA reduces FN more than FP.

**Fig. 5:**
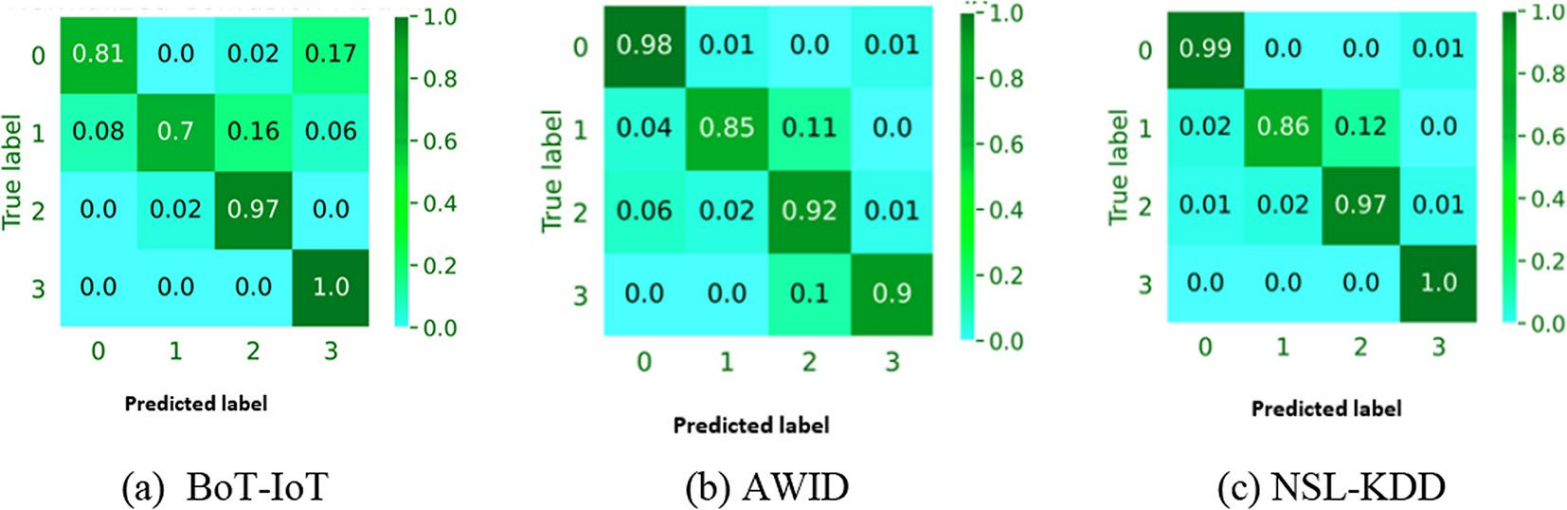
Normalized confusion matrices based on the BoT-IoT, AWID, and NSL-KDD datasets.

Figure 5a–c show how well the FCM-SWA model with a 10-fold CV can classify objects into more than one category for the BoT-IoT, AWID, and NSL-KDD datasets, respectively. The proposed FCM-SWA clearly does not concentrate on a single class, instead recommending the identification of critical features for all classes. However, it cannot guarantee the performance of all attacks, especially those with few dataset instances. Furthermore, the majority of classifications demonstrate satisfactory performance, but several attacks, including “theft attacks” and “reconnaissance” in the AWID dataset, are difficult to classify. This outcome stems from the fact that the total data set contains fewer incidents of these attacks than other types, thereby influencing their classification.

**Table 3:** Label encoding for the BoT-IoT, AWID, and NSL-KDD datasets.

BoT-IoT		AWID		NSL-KDD	
Class	Code	Class	Code	Class	Code
Theft attacks	0	Injection	0	U2R	0
Reconnaissance	1	Impersonation	1	Probe_attacks	1
DoS attacks	2	Flooding	2	DoS_attacks	2
DDoS attacks	3	Normal	3	Normal	3

**Table 4:** The average model accuracy varies with the size of the training dataset.

Dataset	Dataset size	TP (%)	FP (%)
NSL-KDD	100	89.5	0.65
	500	89.9	0.64
	1000	91.0	0.63
	10,000	93.8	0.02
	20,000	93.9	0.02
	80,000	95,22	0,01
AWID	10,000	92.5	0.55
	100,000	92.9	0.57
	200,000	93.0	0.61
	400,000	94.8	0.02
	800,000	95.9	0.01
	1,000000	97.5	0.01
BoT-IoT	10,000	91.3	0.48
	100,000	92.8	0.52
	200,000	94.1	0.57
	400,000	94.6	0.19
	800,000	96.4	0.01
	1,000000	97.1	0.01

**Table 5:** Confusion matrix of 20,000 DoS attacks (BoT-IoT data set).

	Predicted	
Actual	Positive	Negative
Positive	1027	339
Negative	243	18,391

### Result analysis using BoT-IoT dataset

In this subsection, the performance of the proposed FCM-SWA model is evaluated using the BoT-IoT dataset. With the BoT-IoT dataset as its basis, Table 6 shows how well KNN, NB, RF, LR, SVMs, ANN, and the proposed FCM-SWA classification algorithms work. It is evident that FCM-SWA provides the highest DR for DoS attacks and reconnaissance, at 99.65% and 100%, respectively. With FCM-SWA, the DR for theft attacks and DDoS is 96.65% and 97.89%, respectively. We conducted numerous tests on the KNN using various values for k, specifically k = 2, 4, 6, 8, and 10, respectively. KNN performed best at k = 8 and achieved DR values of 93.01% for DDoS, 97.41% for DoS, 97.71% for reconnaissance, and 91.22% for theft attacks. Similarly, the RF classifier has attained DRs of 95.78% for DDoS, 98.25% for DoS, 99.69% for reconnaissance, and 95.23% for theft attacks. Similarly, the LR classifier has a DR of 92.67% for DDoS, 96.25% for DoS, 97.69% for reconnaissance, and 93.2% for theft attacks. Anyone can verify that the suggested FCM-SWA model outperformed all previously deployed classifiers in terms of DR, PR, and F1 scores based on a 10-fold CV. Therefore, the FCM-SWA model can distinguish between true and false IDS alarms.

**Table 6:** Performance evaluation for the proposed FCM-SWA and other existing algorithms in terms of DR, PR, and F1 score using the BoT-IoT dataset (in average—five runs).

Models	Evolution Parameters											
	DR				PR				F1 Score			
	DDoS	DoS	Reconnaissance	Theft	DDoS	DoS	Reconnaissance	Theft attacks	DDoS	DoS	Reconnaissance	Theft
NB	93.4	99.32	99.78	94.2	91.75	99.46	99.93	97.75	91.25	99.39	99.85	97.42
KNN (k = 8)	93.01	97.41	97.71	91.22	85.75	97.6	97.91	95.26	95.2	97.5	97.81	93.59
RF	95.78	98.25	99.69	95.23	89.75	98.06	99.67	93.42	92.25	98.16	99.68	93.34
LR	92.67	96.25	97.69	93.23	87.75	96.06	97.67	91.42	90.25	96.16	97.68	91.34
SVM	95.01	99.41	99.71	93.22	87.75	99.6	99.91	97.26	97.2	99.5	99.81	95.59
ANN	95.4	99.52	100	97.23	91.75	99.86	99.78	95.42	94.25	99.56	99.78	95.34
FCM-SWA	96.65	99.65	100	97.89	93.75	100	99.98	99.75	93.25	100	100	99.42

**Fig. 6:**
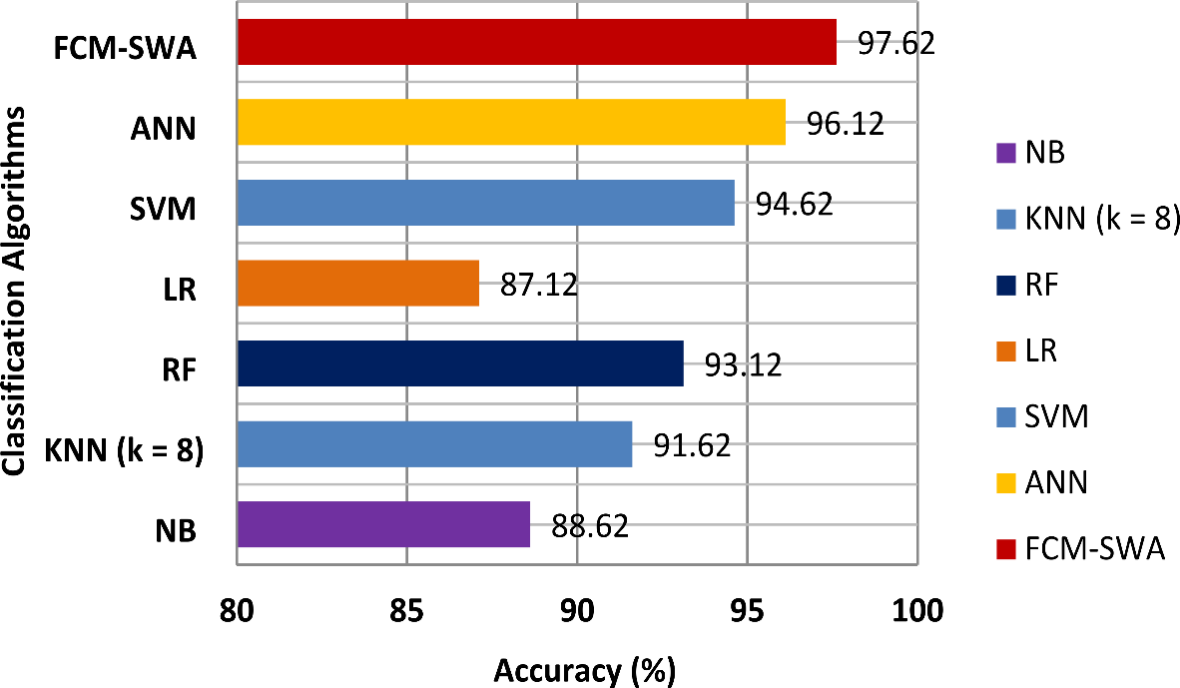
Performance analysis of six classifiers and FCM-SWA in terms of accuracy using the BoT-IoT dataset.

Figure 6 compares the FCM-SWA’s accuracy with that of seven well-known classifiers. It demonstrates that the FCM-SWA significantly outperformed other classifiers in terms of accuracy. Furthermore, we observed that the LR and NB classifiers showed weak reliability, achieving minimal accuracy of only 87.12% and 88.62%, respectively. Both KNN and RF attempted to manage effectively by achieving small increases in accuracy of 91.62% and 93.12%, respectively. Furthermore, the SVM and ANN models produced outcomes closer to FCM-SWA by achieving an accuracy of 94.62% and 96.12%, respectively. However, the presented FCM-SWA algorithm produced greater results, achieving an accuracy of 97.62%.

**Fig. 7:**
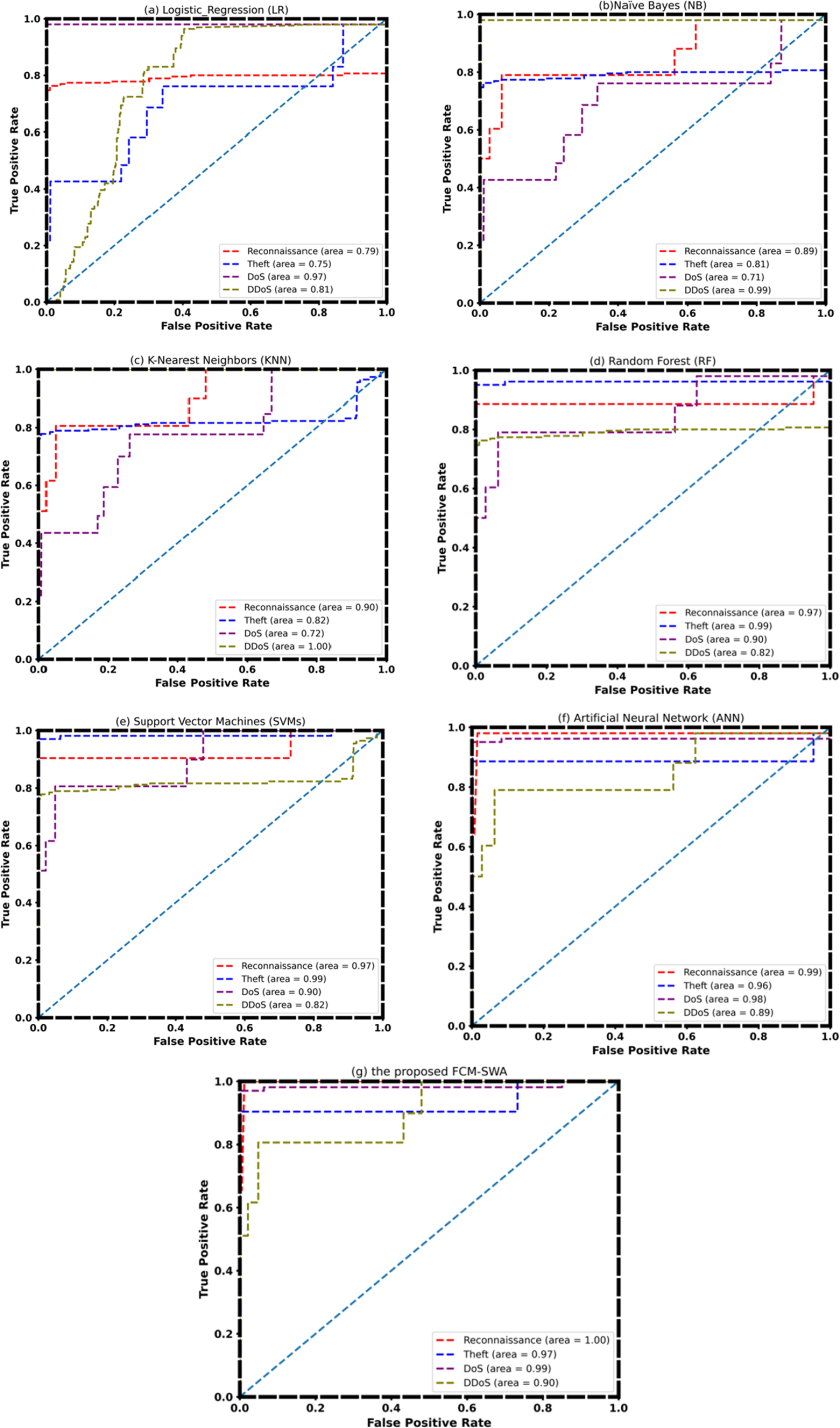
ROC Curve of**a** Logistic Regression (LR), **b** Naïve Bayes (NB), **c** K-Nearest Neighbors (KNN), **d** Random Forest (RF), **e** Support Vector Machines (SVMs), **f** Artificial Neural Network (ANN), and **g** the proposed FCM-SWA using BoT-IoT dataset.

Figure 7 depicts the receiver operating characteristic (ROC) curves for all classification models. We can regard SVM and ANN as more accurate because the area under the curves (AUC) for the classifications of reconnaissance, theft attacks, and denial of service are all close to or near one. In the cases of RF and KNN, the AUC only approaches unity for DDoS and theft attacks. This demonstrates that the KNN model distinguishes DDoS attacks from normal traffic effectively. On the other hand, KNN is a superior algorithm for DDOS attacks. However, with FCM-SWA, the effectiveness of reconnaissance, DoS, and theft attacks is almost equal.

### Result analysis using NSL-KDD dataset

This subsection evaluated the performance of the suggested FCM-SWA using the real NSL-KDD dataset. Table 7 presents the results of the class-wise predictions for LR, NB, KNN, RF, SVMs, ANN, and the proposed FCM-SWA. It is clear that FCM-SWA outperformed LR, NB, KNN, RF, SVMs, and ANN in terms of attack detection rate. The LR-based detection system has a detection rate of 93.79% for normal, 91.19% for DoS attacks, 91.4% for probe attacks, and 89.77% for U2R. The detection system based on KNN has demonstrated the best performance at k = 8 and gained a DR of 95.81% for normal, 93.21% for DoS attacks, 93.51% for probe attacks, and 91.02% for U2R. Similarly, the RF classifier has obtained DR values of 96.82% for normal, 94.22% for DoS attacks, 94.72% for Probe attacks, and 92.79% for U2R. The FCM-SWA classifier has a DR of 99.74% for normal, 97.25% for DoS attacks, 98.14% for probe attacks, and 95.78% for U2R. The suggested FCM-SWA model also did better than all the other classifiers in terms of DR, PR, and F1 score, as shown by an empirical analysis of their success rates. On the other hand, the LR-based detection system has the lowest ratings for DR, PR, and F1.

**Fig. 8:**
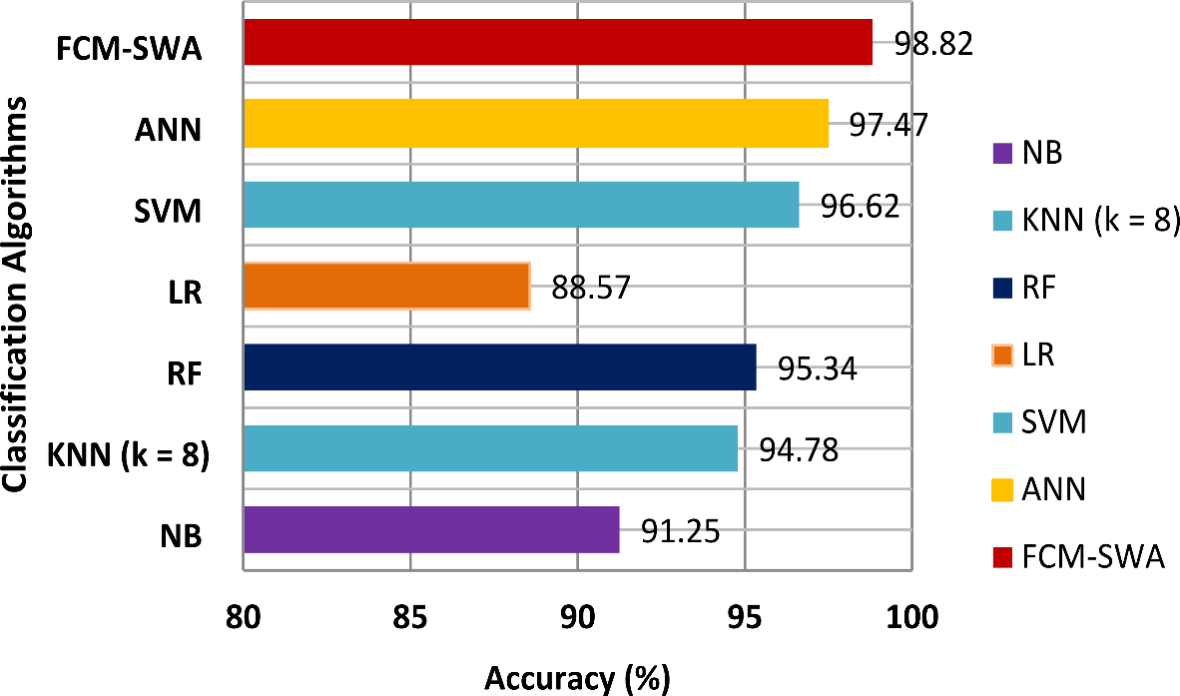
Performance analysis of all classification algorithms and FCM-SWA in terms of accuracy using the NSL-KDD dataset.

Again, as demonstrated in Fig. 8, the FCM-SWA classifier performed exceptionally well in terms of accuracy compared to other approaches. FCM-SWA has an accuracy of 98.82%, whereas NB has the worst performance with an accuracy of 88.57%. Simultaneously, both the NB and KNN classifiers attempted to perform well, achieving a slight increase in accuracy of 91.25 and 94.78%, respectively. ANN and SVM classifiers provided results closer to FCM-SWA, with accuracy of 97.47% and 96.62%, respectively.

**Fig. 9:**
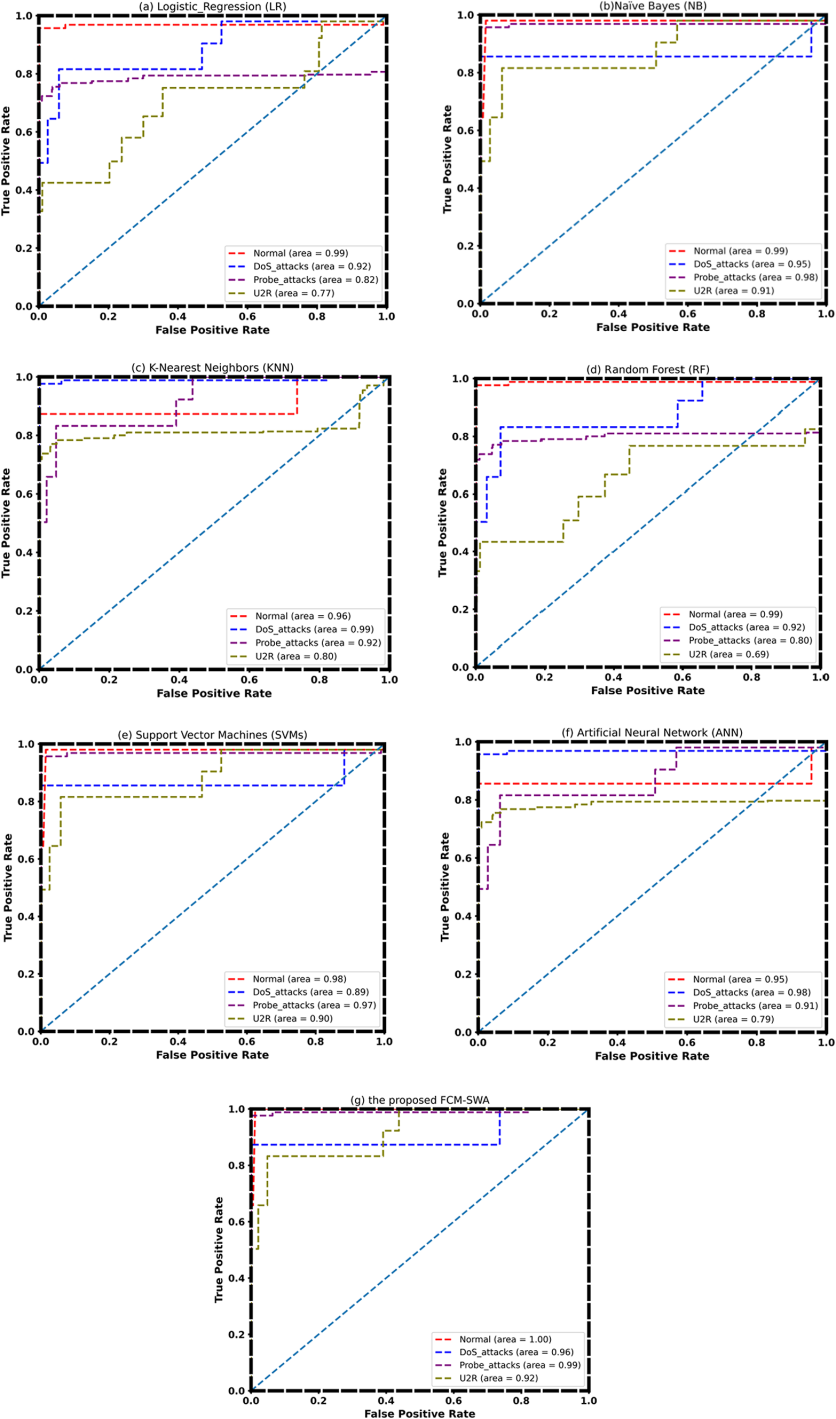
ROC Curve of**a** Logistic Regression (LR),**b** Naïve Bayes (NB),**c** K-Nearest Neighbors (KNN),**d** Random Forest (RF),**e** Support Vector Machines (SVMs),**f** Artificial Neural Network (ANN), and (g) the proposed FCM-SWA using NSL-KDD Dataset.

Finally, the ROC curve for all classification models over the full feature space is shown in Fig. 9. When we compare the area under the curves for each class to the value 1, we notice that the area under the curves of FCM-SWA is one or close to one for probe_attack, DoS, U2R, and normal. However, the area under the curve for SVM and NB only approaches the one for probe_attack and normal.

**Table 7:** Performance evaluation for the proposed FCM-SWA and other existing algorithms in terms of DR, PR, and F1 score using the NSL-KDD dataset (in average—five runs).

Models	Evolution Parameters											
	DR				PR				F1 Score			
	Normal	DoS_attacks	Probe_attacks	U2R	Normal	DoS_attacks	Probe_attacks	U2R	Normal	DoS_attacks	Probe_attacks	U2R
NB	94.80	92.20	92.50	90.01	89.23	94.22	91.33	94.05	90.44	93.99	91.23	92.38
KNN (k = 8)	95.81	93.21	93.51	91.02	90.24	95.23	92.34	95.06	91.45	95.00	92.24	93.39
RF	96.82	94.22	94.72	92.79	91.25	96.24	93.35	96.07	92.46	96.01	93.25	94.40
LR	93.79	91.19	91.49	89.77	95.38	83.53	95.69	93.04	95.28	92.98	95.59	91.37
SVM	97.83	95.23	95.83	93.56	92.26	97.25	94.36	97.08	93.47	97.02	94.26	95.41
ANN	98.84	96.24	96.64	94.13	99.86	98.26	99.35	98.09	99.75	98.03	99.89	96.42
FCM-SWA	99.74	97.25	98.14	95.78	100.00	99.89	100.00	99.10	100.00	99.04	99.94	97.43

### Result analysis using AWID dataset

In this section, we used the IoT-based AWID dataset to investigate the proposed model’s performance. Table 8 displays the results of class-wise predictions for seven classifiers. The suggested FCM-SWA model outperformed LR, NB, KNN, RF, SVMs, and ANN approaches in terms of attack detection rate. Similarly, in terms of precision rate and F1 score, FCM-SWA is superior to other existing classification techniques. The precision rate based on LR has achieved 97.38% for normal, 92.53% for DoS attacks, 97.69% for probe attacks, and 95.04% for U2R. Similarly, the RF classifier has attained a precision rate of 93.25% for normal, 98.24% for DoS attacks, 95.35% for probe attacks, and 98.07% for U2R. The FCM-SWA classifier has obtained a 100% precision rate for normal, 100% for DoS attacks, 100% for probe attacks, and 100% for U2R. The FCM-SWA classifier outperformed the other available classification models in terms of DR, PR, and F1 score. Unfortunately, LR generates the worst results.

**Table 8:** Performance evaluation for the proposed FCM-SWA and other existing algorithms in terms of DR, PR and F1 score using AWID dataset (in average—five runs).

Models	Evolution parameters											
	DR				PR				F1 Score			
	Normal	DoS_attacks	Probe_attacks	U2R	Normal	DoS_attacks	Probe_attacks	U2R	Normal	DoS_attacks	Probe_attacks	U2R
NB	94.45	94.2	94.5	93.01	91.23	95.22	94.33	95.05	92.44	95.99	93.23	94.38
KNN (k = 8)	95.02	95.21	95.51	93.02	92.24	97.23	94.34	97.06	93.45	97	94.24	95.39
RF	96.33	96.22	96.72	94.79	93.25	98.24	95.35	98.07	94.46	98.01	95.25	96.4
LR	93.79	93.19	93.49	91.77	97.38	92.53	97.69	95.04	97.28	94.98	97.59	93.37
SVM	97.41	97.23	97.83	95.56	94.26	99.25	96.36	99.08	95.47	99.02	96.26	97.41
ANN	98.02	98.24	98.64	96.13	98.86	100	99.56	100	99.78	98.25	99.58	98.42
FCM-SWA	98.74	99.25	99.14	97.78	100	100	100	100	100	100	100	99.43

**Fig. 10:**
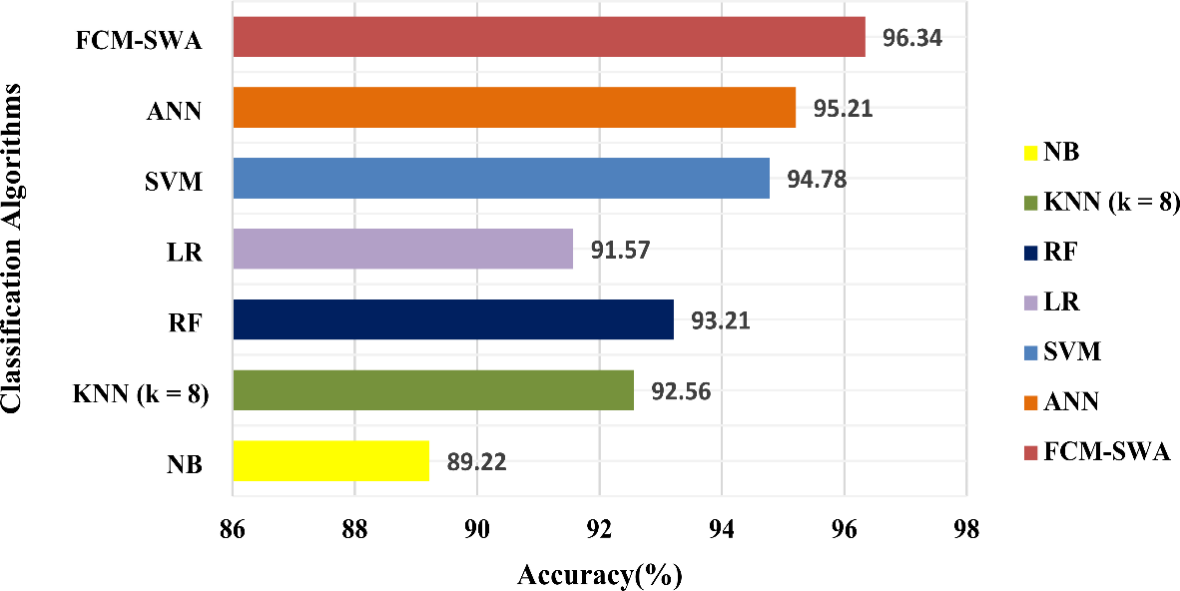
Performance analysis of all classification algorithms and FCM-SWA in terms of accuracy using AWID Dataset.

**Fig. 11:**
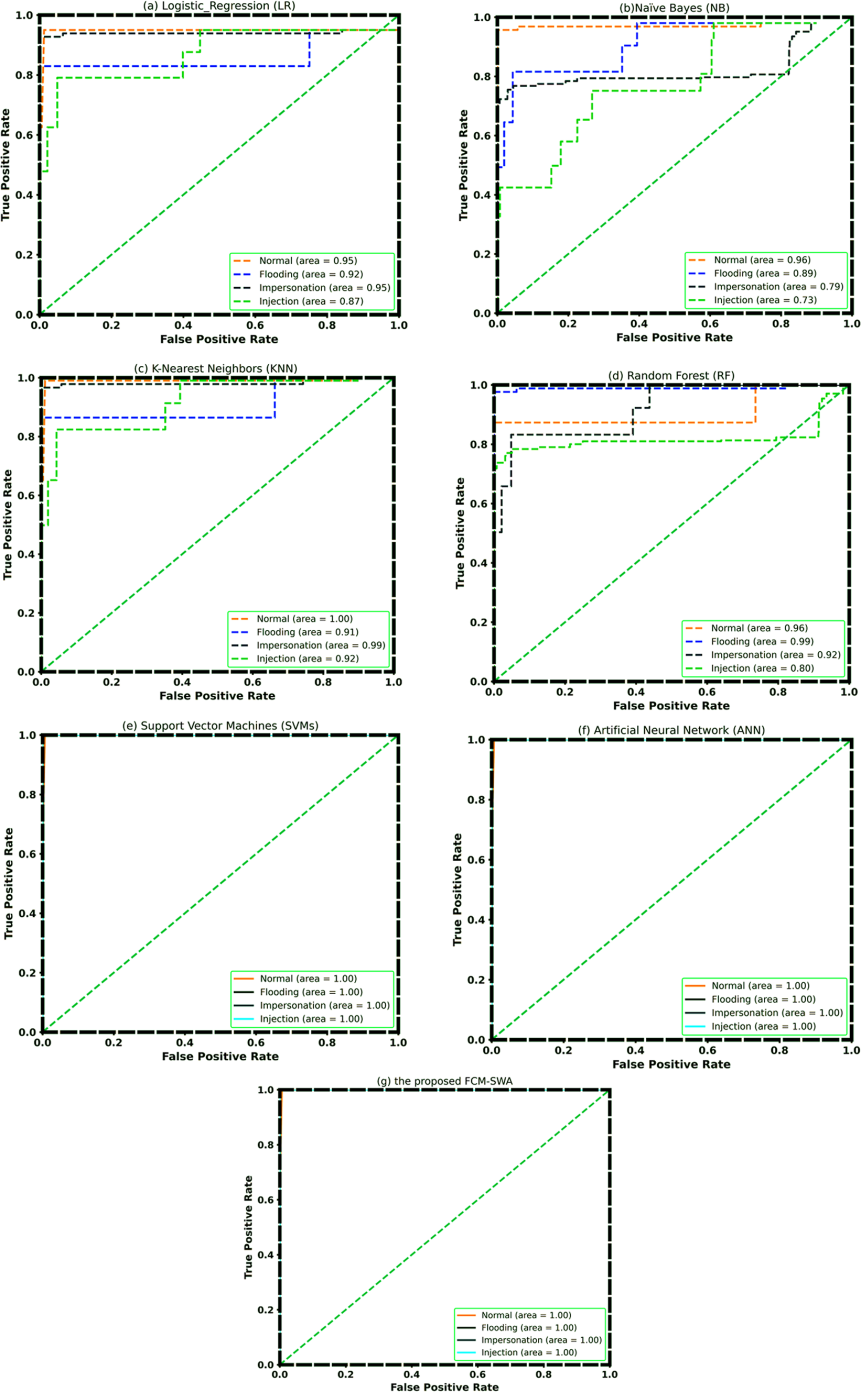
ROC Curve of**a** Logistic Regression (LR), **b** Naïve Bayes (NB), **c** K-Nearest Neighbors (KNN), **d** Random Forest (RF), **e** Support Vector Machines (SVMs), **f** Artificial Neural Network (ANN), and **g** the proposed FCM-SWA using AWID Dataset.

Figure 10 depicts a comprehensive accuracy comparison between seven classifiers and the proposed FCM-SWA model. It is clear that the models ANN, SVM, RF, KKN, NB, and FCM-SWA achieved 95.21%, 94.78% and 96.34%, respectively. In general, the FCM-SWA significantly outperformed other models in terms of accuracy. Finally, Fig. 11 depicts the ROC curve for all classification models utilizing the AWID dataset. It can be seen from ROC that FCM-SWA, SVM, and ANN have the highest accuracy. Because the area under the curves of each class equals one.

### Comparison with the state of the art methods

Table 9 presents a comparison of various models applied to different datasets for intrusion detection, showing their respective accuracy rates across three datasets: NSL-KDD, AWID, and BoT-IoT. For the NSL-KDD dataset, Diro & Chilamkurti presented a deep learning model that achieved 96.50% accuracy, while Pajouh et al. presented a two-tier model that recorded 85.24%. Other models like RNN and MDPCA showed lower accuracy rates of 81.29% and 82.08%, respectively. Gao et al. introduce an adaptive ensemble classifier, and Tian et al. propose a DBN model that achieved 85.79% and 96.17%, respectively, while Biazar et al. present a MLP-SSO model that reached 95.78%. In comparison, the proposed FCM-SWA model outperformed all of these models, achieving a notable accuracy of 98.82%.

For the AWID dataset, Alotaibi & Elleithy proposed a voting ensemble model that recorded 96.32%, followed closely by Kolias et al. at 96.20%. Wang et al. demonstrated lower accuracy, achieving 92.49%. Biazar et al. presented an MLP-SSO model with an accuracy of 94.23%, while the proposed FCM-SWA model outperformed the others with a slightly better result of 96.34%.

In the BoT-IoT dataset, Susilo and Sari introduced a CNN model that achieved 91.27%, and Ibitoye et al. proposed a feedforward neural network that reached 95.1%. Additionally, Biazar et al. presented an MLP-SSO model that achieved a score of 96.31%. Once again, the proposed FCM-SWA model demonstrated superior performance, achieving the highest accuracy of 97.62%, indicating its effectiveness across all datasets.

**Table 9:** Comparison results with other existing methods on NSL-KDD, AWID, and BoT-IoT.

Ref	Classifiers	Dataset	Accuracy (%)
Diro & Chilamkurti [15]	Deep Learning (DL)	NSL-KDD	96.50
Pajouh et al. [18]	Two-Tier model	NSL-KDD	85.24
Wu et al. [48]	RNN	NSL-KDD	81.29
Yang et al. [51]	MDPCA	NSL-KDD	82.08
Gao et al. [46]	adaptive ensemble classifier	NSL-KDD	85.79
Tian et al. [50]	DBN	NSL-KDD	96.17
Biazar et al. [54]	MLP-SSO	NSL-KDD	95.78
The proposed model	FCM-SWA	NSL-KDD	98.82
Alotaibi & Elleithy [57]	Voting(ET, RF, Bagging)	AWID	96.32
Kolias et al. [33]	J48	AWID	96.20
Wang et al. [40]	DNN	AWID	92.49
Biazar et al. [54]	MLP-SSO	AWID	94.23
The proposed model	FCM-SWA	AWID	96.34
Susilo and Sari [55]	Convolutional neural network (CNN)	BoT-IoT	91.27
Ibitoye et al. [56]	Feedforward Neural Networks (FNN)	BoT-IoT	95.1
Biazar et al. [54]	MLP-SSO	BoT-IoT	96.31
The proposed model	FCM-SWA	BoT-IoT	97.62

## Conclusions

This paper develops a novel approach, FCM-SWA, to safeguard IoT-based smart networks against botnet attacks. Three phases comprise the proposed approach: the data preprocessing phase, feature mapping, and clustering phase. The initial steps include preprocessing incoming IoT network data, mapping features, and normalizing them to select relevant features from the networks of smart cities. We then send the data to the subsequent phase, which uses the MCC approach for feature ranking and selection. The final step utilizes a hybrid FCM-SWA algorithm for feature selection, identification, and decision-making. To increase classification accuracy, we employ FSM-SWA, a hybrid approach that combines FCM and SWA, to choose a subset of the original features. We train and verify classifiers such as NB, RF, LR, SVMs, ANNs, and the proposed FCM-SWA approach on three datasets. The suggested approach outperformed the other classifiers in terms of F-measure, sensitivity, recall, specificity, and accuracy.

## Data Availability

NSL-KDD dataset: https://web.archive.org/web/20150205070216/http://nsl.cs.unb.ca/NSL-KDD/AWID dataset: https://icsdweb.aegean.gr/awid/download-dataset BoT-IoT dataset: https://research.unsw.edu.au/projects/bot-iot-dataset.
